# Synthesis and Characterization of Piano-Stool Ruthenium(II)–Arene Complexes of Isatin Schiff Bases: Cytotoxicity and DNA Intercalation

**DOI:** 10.1021/acsomega.3c10265

**Published:** 2024-04-17

**Authors:** Hande Karabıyık, Aslıhan Karaer Tunçay, Suleyman Ilhan, Harika Atmaca, Hayati Türkmen

**Affiliations:** † Faculty of Science, Department of Physics, Dokuz Eylül University, Izmir 35390, Turkey; ‡ Department of Chemistry, Faculty of Science, Ege University, Bornova, Izmir 35100, Turkey; § Department of Biology, Faculty of Engineering and Natural Sciences Manisa Celal Bayar University, Manisa 45140, Turkey

## Abstract

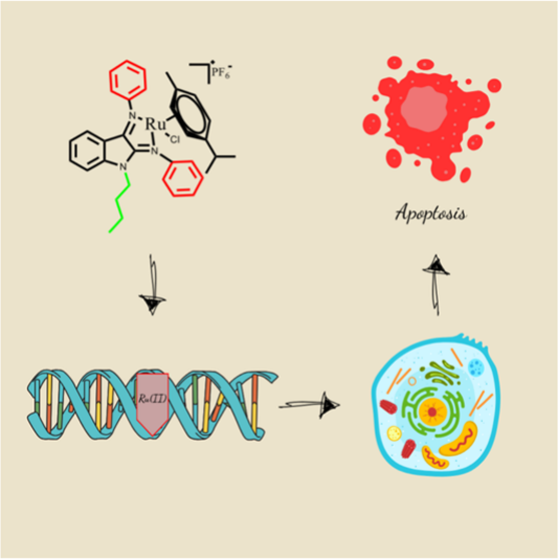

A series of aryl–isatin Schiff base derivatives (**3a**–**d**) and their piano-stool ruthenium complexes (**4a**–**d**) were synthesized and characterized via ^1^H and ^13^C NMR and Fourier transform infrared (FTIR) spectroscopy. In addition, the purity of all of the compounds (**3a**–**c** and **4a**–**d**) was determined via elemental analysis. Complex **4d** was analyzed using X-ray crystallography. An *in vitro* antiproliferative study of the compounds (**3a**–**c** and **4a**–**d**) against human hepatocellular carcinoma (HEPG2), human breast cancer (MCF-7), human prostate cancer (PC-3), and human embryonic kidney (HEK-293) cells exhibited their considerable antiproliferative activity. **4d** exhibited effective cytotoxicity against HEPG2 and MCF-7. It displayed higher cytotoxicity than the reference metallo-drug cisplatin. Moreover, the stability of **4d** was studied via ^1^H NMR spectroscopy, and the binding model between **4d** and DNA was investigated via ultraviolet–visible spectroscopy. The lipophilicity of the synthesized complexes was determined using an extraction method.

## 1 Introduction

Cancer is one of the most significant health problems in recent times worldwide. Its traditional treatments include surgery, radiotherapy, and chemotherapy. Thus far, the lack of a fully curative treatment for most types of cancer has led to the development of new therapeutic agents. Platinum-based drugs, such as cisplatin, carboplatin, and oxaliplatin, have been among the most effective chemotherapeutic agents for the treatment of carcinomas for years. But, the high toxicity or incidence of acquired drug resistance limits the clinical use of these platinum-derived drugs.^1,2^ In the last two decades, ruthenium compounds have attracted considerable interest as potential anticancer agents because of their low toxicity, efficacy against platinum drug-resistant tumors, and high selectivity for tumors, and their preclinical and early clinical trials have provided promising results at different stages.^3^ In addition, the unique properties of ruthenium-based drugs, such as slow ligand exchange rates, a range of oxidation states [Ru(II), Ru(III) and Ru(IV)], and the ability to overcome platinum resistance, show promise for the use of ruthenium compounds in cancer therapy.^4,5^ The ruthenium-based chemotherapeutic derivatives NAMI-A, KP1019, NKP1339, and TLD1443 exhibit *in vitro* and *in vivo* activities.^6^ Thus far, clinical trials of the two ruthenium complexes [ImH]trans-[RuCl_4_(Im)(dmso-S)], (NAMI-A, Im = imidazole, dmso = dimethyl sulfoxide) and [IndH]*trans*-[RuCl_4_(Ind)_2_], (KP1019, Ind = indazole) have been completed.^7,8^ However, interaction of ruthenium complexes with DNA has received notable attention after Clark’s study. Chelating compounds are used to mediate duplex DNA helix scission of anticarcinogenic agents.^9^ In 2019, Puthilibai and Vasudhevan synthesized a novel octahedral Ru(II) isatin complex, *cis*-[Ru(Phen)_2_ FPIMI] ClO_4_·2H_2_O via reactions between *cis*-[Ru (Phen)_2_Cl_2_]·2H_2_O and 4-fluoro phenyl imino methyl isatin (FPIMI). DNA intercalation interactions, an *in vitro* anticancer study, and cytotoxic activities of the isatin-based Ru(II) Schiff base complex were investigated. The IC_50_ value exhibited more potent *in vitro* cytotoxic activity against selected human cell lines compared with the newly synthesized ruthenium(II) complex ligand (26 ± 0.5 μM). Furthermore, the anticancer activity of the complex was four times more potent than that of cisplatin, with less cytotoxicity against selected human tumor cell lines. Although the intrinsic binding constant (*K*_b_) of the complex with DNA was lower than that of typical intercalators, the synthesized isatin-based Ru(II) complex had less cytotoxicity.^10^ Recently, our group synthesized a series of mono- and bimetallic Ru(II)–arene complexes and investigated their anticancer properties on HeLa, MDA-MB-231, DU-145, LNCaP, HEPG2, Saos-2, PC-3, and MCF-7 and the normal cell lines 3T3-L1 and Vero.^11^ İnan et al. reported cytotoxic activities for a series of new Ru(II) complexes containing the N–N group synthesized from (*E*)-2-hydroxy-5-(phenyldiazenyl)benzaldehyde-based Schiff base ligands. The antiproliferative activities of Schiff base ligands and their Ru(II) complexes were investigated *in vitro* in H2126, PC-3, and MCF-7 cancer cell lines. Ruthenium complexes exhibited low to moderate *in vitro* antiproliferative activities in selected cell lines compared to the drug 5-FU as a positive control.^12^ Kumar et al. reported the synthesis of six new half-sandwich Ru(II) complexes of the type [Ru(η6-arene)(L)Cl](arene = benzeneorp-cymene; L = 1-pyrenecarboxaldehyde benzhydrazone ligands). Anticancer activities of Ru(II) complexes were determined using cisplatin as a positive control against MCF-7 and A549 and NIH 3T3 by measuring cell viability via colorimetric analysis. New ruthenium–arene benzhydrazone complexes showed that their cytotoxicity toward A549 cells was significantly superior to that of cisplatin.^13^

Lipophilicity is a notable factor in examining the pharmacokinetic properties of a drug and its interactions with macromolecular targets. Lipophilic molecules can pass easily through cell membranes. The partition coefficient between water and *n*-octanol is generally measured and expressed as log*P* in a bid to measure the affinity of a molecule for a lipophilic environment.^14^ The partition coefficient is an important notion in structure–activity relationship (SAR) and quantitative structure–activity relationship studies.^15^

We report the synthesis of novel Ru(II) complexes bearing different isatin Schiff bases and their effects on the following cell lines: human hepatocellular carcinoma (HEPG2), human breast cancer (MCF-7), human prostate cancer (PC-3), and human embryonic kidney (HEK-293). We also discuss the lipophilic properties of these complexes to characterize the relationship between their structure and activity. *In vitro* testing showed that the most lipophilic complex **4d** had the greatest cytotoxic activity. We investigate the effects of aryl ligand types and *N*-alkylated ligand types on Ru(II)arene complexes in cytotoxic activity studies. All complexes exhibited enhanced activity compared with that of the parent ligand. The lipophilicity of the complexes is increased by the addition of an alkyl substituent to the nitrogen of the isatin ligand. Overall, Ru complexes show promise as potential anticancer drugs. To overcome the limitations associated with Pt-based chemotherapeutic drugs, Ru complexes can be used as an alternative, owing to their mentioned unique properties.

## 2 Results and Discussion

### 2.1 Chemistry

**Scheme 1:**
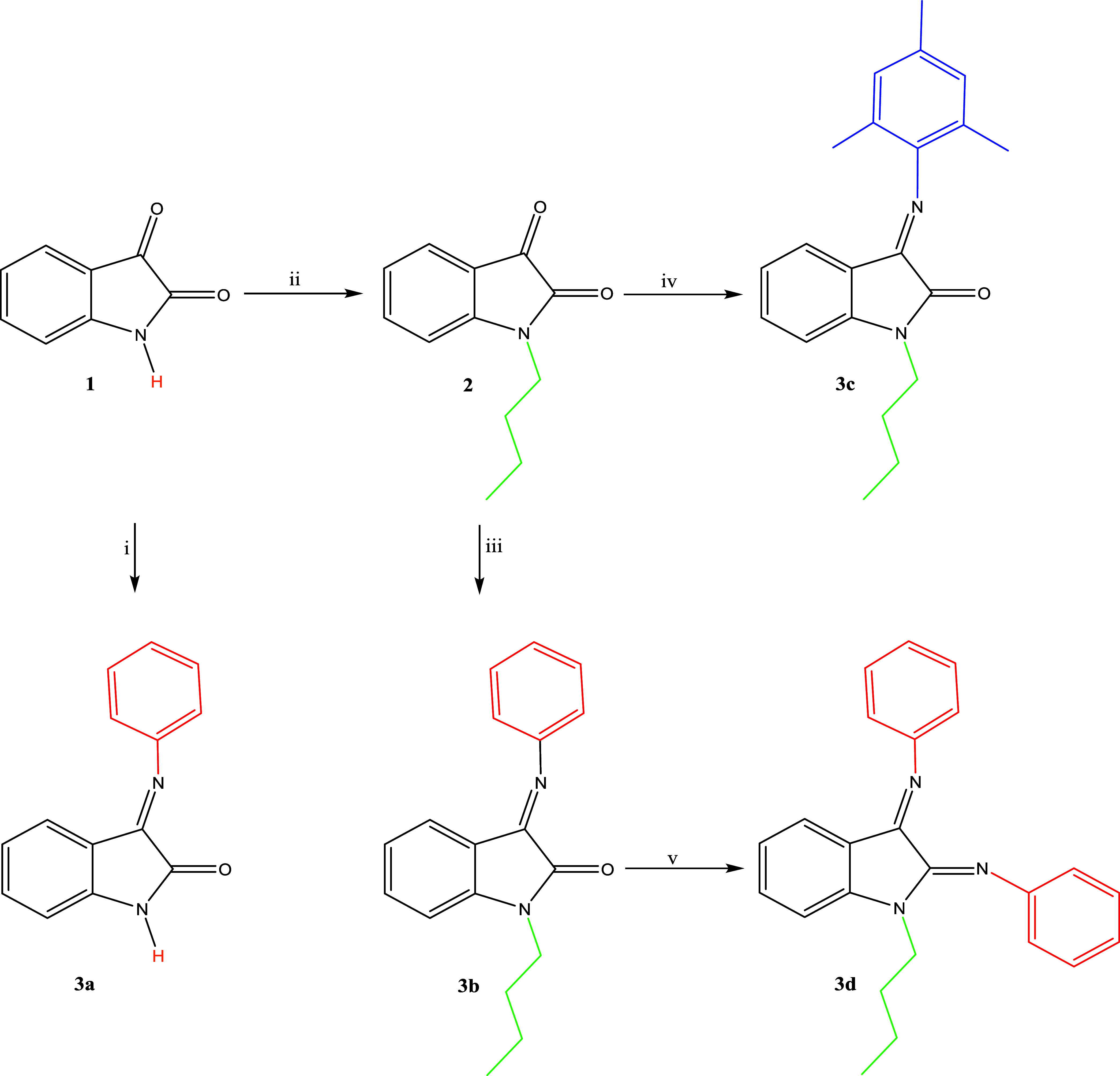
Synthesis of Ligands **3a**–**d** Reaction conditions: (i) aniline, EtOH, 50 °C, 6 h, 85% yield; (ii) 1-bromobutane, K_2_CO_3_, DMF, 70 °C, 2 h, 74% yield; (iii) aniline, 50 °C, 6 h, 65% yield; (iv) 2,4,6-trimethylaniline, EtOH, 50 °C, 24 h, 86% yield; and (v) aniline, TiCl_4_, NEt_3_, toluene, 90 °C, 2 h.

Isatin Schiff base derivatives have been synthesized to obtain effective and selective medicinal agents with various substituents in any part of the skeletal structure. The wide-ranging applications of isatin derivatives are associated with their versatility, allowing for the construction of a variety of structures suitable for a particular reactivity or chemical property of interest. Hence, we prepared isatin Schiff bases containing aryl and alkyl groups and their ruthenium(II)–arene complexes to investigate their anticancer activities. The aromatic ring on the compound provided a steric effect and a hydrophobic surface, and the hydrocarbon chain group (alkyl) afforded solubility in oil. This study aimed to investigate the effects of the aryl group on the isatin Schiff bases and ligand properties of their complexes on cytotoxic activity. Scheme 1 shows the synthesis pathways of the isatin Schiff bases. In the first step, *N*-butylisatin (**2**) was prepared via the reaction of isatin with bromobutane in the presence of K_2_CO_3_ in dimethylformamide (DMF).^16^ The monoaryl-based isatin Schiff bases **3a**–**c** were readily accessible in satisfactory yield from *N*-butylisatin (**2**) by means of heating with aniline and 2,4,6-trimethylaniline in EtOH separately. For comparison, compound (**3a**) was directly prepared from isatin and aniline in 1:1 stoichiometry in EtOH. The diaryl-based isatin Schiff base (**3d**) was designed and prepared, but featuring **3d** failed under various reaction conditions, and only insoluble or intractable mixtures could be obtained. Isatin Schiff bases (**3a**–**c**) were purified via recrystallization using ethanol. They were soluble in chlorinated solvents, alcohols, and DMSO. The infrared spectra of **3a**–**c** exhibited many bands of varying intensities within the range of 400–4000 cm^–1^. Assignment of each individual band to a specific vibration was not attempted. The −C=N group in the isatin Schiff bases was confirmed with the observation of ν(C=N) bands between 1646 and 1665 cm^–1^. All ligands (**2**, **3a–c**) and complexes **4b**–**d** were examined via proton (^1^H) nuclear magnetic resonance (NMR) in CDCl_3_. However, **4a** was examined in DMSO because of the former’s low solubility in chloroform. The ^1^H NMR spectra of **3a** showed a signal owing to the NH group of isatin at 9.83 ppm. In the ^1^H NMR spectrum for **3b** and **3c**, aliphatic CH_3_ protons of *n*-butyl were observed as triplets at 0.98 and 0.99 ppm, respectively. The ^1^H NMR spectrum of **3b** exhibited a singlet resonance at 2.32 and 1.99 ppm because of methyl groups at the 2,4,6-position of the mesityl ring. All other spectral data agreed with the assumed structures.

**Scheme 2:**
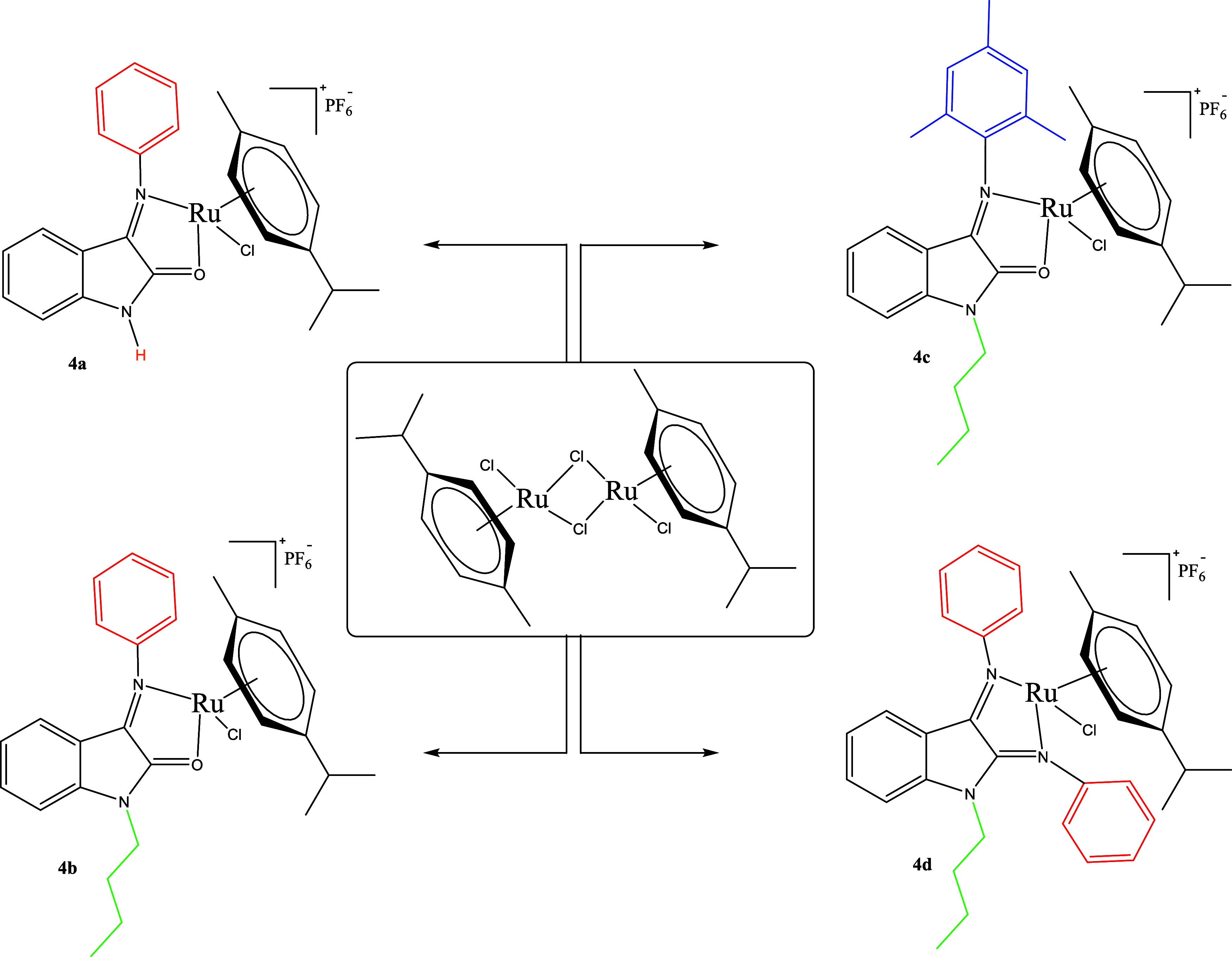
Synthesis of Complexes **4a–d** Reaction conditions: (a) [RuCl_2_(*p*-cymene)]_2_, chloroform, room temperature (RT) 6 h, 40% yield; (b) [RuCl_2_(*p*-cymene)]_2_, chloroform, RT 6 h, 44% yield; (c) [RuCl_2_(*p*-cymene)]_2_, chloroform, 70 °C, 12 h, 45% yield; and (d) [RuCl_2_(*p*-cymene)]_2_, dichloromethane, RT 6 h, 61% yield.

Scheme 2 shows the synthesis of Ru complexes **(4a–d)**. Ru(II) of isatin Schiff base complexes **4a**–**d** have a cationic structure, which is resistant to air and moisture and forms a chelate complex. The Ru(II)–arene complexes (**4a**–**c**) were synthesized via the reaction of [{(η^6^-*p*-cymene)Ru(μ-Cl)Cl}_2_] with ligands (**3a**–**c**) in CHCl_3_. Notably, complex **4d** was synthesized via reaction with [{(η^6^-*p*-cymene)Ru(μ-Cl)Cl}_2_] *in situ*, without the isolation of ligand **3d** in CH_2_Cl_2_. The stability of complexes **4a**–**d** was ensured by the PF_6_^–^ anion. After separation via crystallization in a dichloromethane (DCM)/diethyl ether system, each desired complex was obtained in high purity as an orange solid. These complexes (**4a**–**d**) can be stored in air for an extended period and are soluble in DMSO, DMF, and MeOH; however, they are insoluble in apolar solvents, such as hexane.

**Figure 1:**
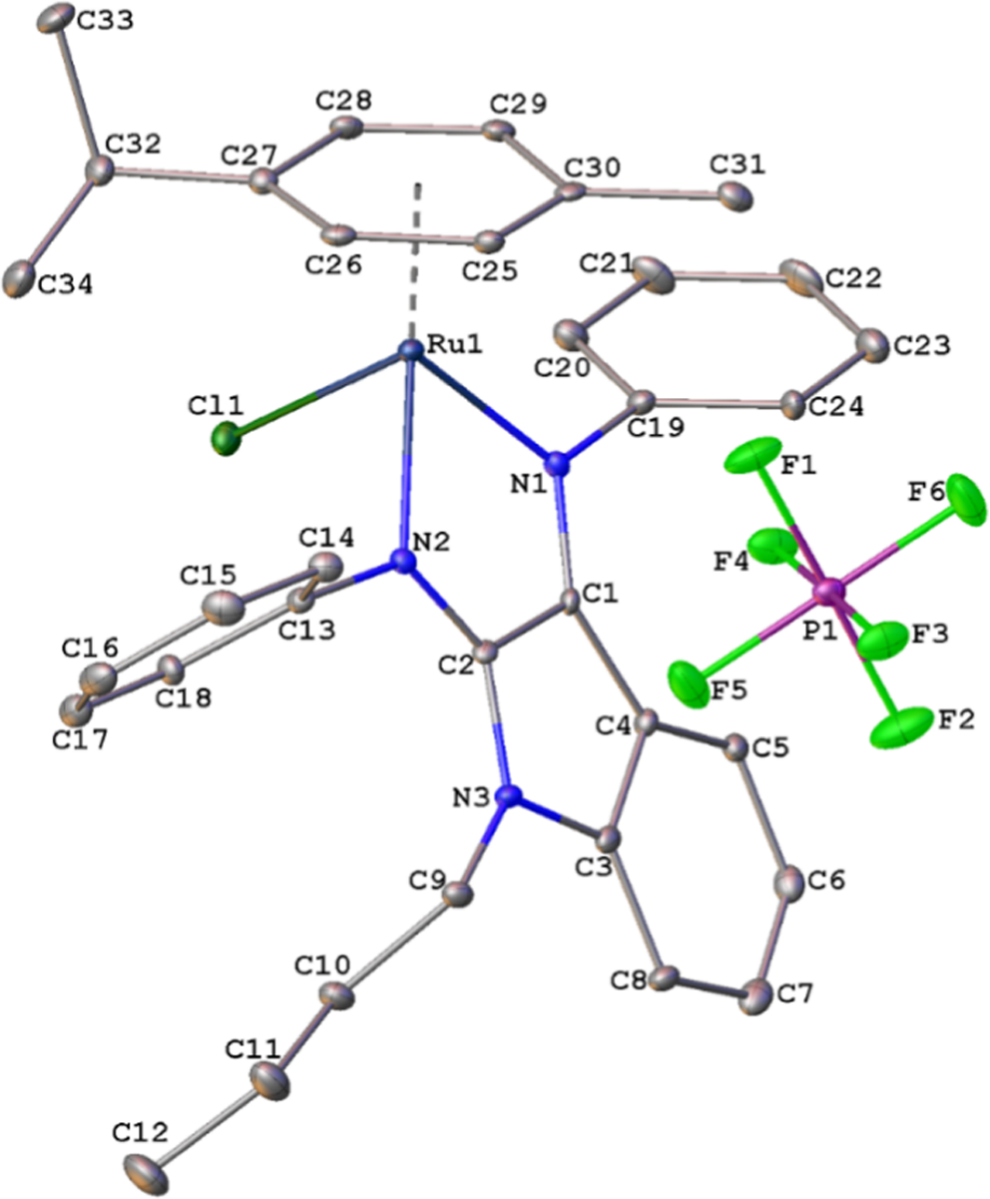
Molecular structure of complex **4d** with thermal ellipsoids plotted at the 30% probability level. Hydrogen atoms are omitted for clarity. Selected bond distances (Å) and angles (deg): Ru1―Cl1 2.446(3), Ru1―N1 2.075(7), Ru1―N2 2.139(7), N2―C2 1.292(10), N1―C1 1.313(10), N2―Ru1―Cl1 85.08(19), N1―Ru1―Cl1 86.74(19), and N1―Ru1―N2 76.5(3).

Almost all complexes (**4a**–**d**) were dissolved to ∼20 mg/mL in DMSO. The complexes were characterized using ^1^H, ^13^C, ^19^F, and ^31^P NMR spectroscopies, Fourier transform infrared (FTIR) spectroscopy (all of structures), and elemental analysis (**3a**–**c**, **4a**–**d**). The FTIR analysis results for the complexes are provided in the Supporting Information (Figures S5, S8, S11, S14, S19, S24 and S29). The typical FTIR of the control, indole, showed an N–H stretching vibration band at 3406 cm^–1^.^17^ The fact that the N–H stretching vibration band of **4a** was still observed at 3411 cm^–1^ indicates that ruthenium did not coordinate via the NH bond. The complexes exhibited bands in the 3000–3200 and 800–850 cm^–1^ regions, which were assumed to indicate C–H vibrations. C–N and C=C vibrations were observed in the regions of 1000–1250 and 1600–1650 cm^–1^, respectively. The NMR spectra of the synthesized compounds were in agreement with the proposed structures. In the ^1^H NMR spectra, the *p*-cymene aromatic peaks of **4d** were observed as four doublets with one proton each. In complexes **4a**–**d**, *p*-cymene aromatic peaks were observed at 6.70–6.32, 6.00–5.55, 6.04–5.39, and 5.29–4.82 ppm, respectively. In addition, the ^1^H NMR spectra of complexes **4a**–**d** exhibited a multiplet at 7.57–6.89, 7.88–6.54, 7.51–6.33, and 7.85–6.56 ppm corresponding to aromatic protons, respectively. The chemical shifts of these protons clearly indicated that **4d** contains the highest number of electron-donating ligands originating from two phenyl rings, and we can say that the electron density of the metal is higher in this complex than in the other complexes. Butyl peaks of the *N*-alkylated complexes (**4b**–**d**) were observed between 0.65 and 3.82 ppm. The ^13^C NMR spectrum of complexes **4a**–**c** exhibited a peak at 184.82, 172.42, and 171.94 ppm, corresponding to the carbonyl C=O carbon, respectively. Single crystals of the solid-state structures of the complexes were obtained by diffusion of diethyl ether into their concentrated solutions in DCM. The molecular structure of **4d** was determined via single-crystal X-ray diffraction. Complex **4d** exhibited a piano-stool type geometry. Figure 1 shows the molecular structure of **4d** along with its atom numbering scheme. The asymmetric unit of **4d** contained one molecule and one hexafluorophosphate anion. Figure 1 shows the selected bond distances and angles for **4d**. Bond distances of Ru1 between C atoms of the C25/C30 ring were in the range of 2.181(8)–2.251(8) Å, whereas the distance between the ring centroid and Ru1 was 1.713(4) Å. Considering the coordination environment, one can see that the bond lengths agree with those in similar structures: the Ru―N and Ru―Cl bond lengths are in the ranges 2 144(10)–2 205(10) and 2.409(4)–2.415 (2) Å, respectively.^18^ In the absence of classical hydrogen bonds, the crystal structure of **4d** is stabilized via π···π stacking interaction between the C3/C8 rings at (*x*, *y*, *z*) and (1–*x*, 1–*y*, 1–*z*) (see Figure S32). The distance between ring centroids was 3.702(6) Å.

All non-hydrogen atoms were anisotropically refined. The positions of the hydrogen atom were geometrically calculated and then refined using the riding model, determining the aromatic C―H distances at 0.93 Å, methylene C―H distances at 0.97 Å, methine C―H distances at 0.98 Å, and methyl C―H distances at 0.96 Å. The *U*_iso_(H) values were set to 1.2*U*_eq_ (1.5*U*_eq_ for the methyl group) of the parent atom. Images of the molecular structure were created using Olex2.^19^ The supplementary crystallographic data for **4d** are provided in the CCDC: 2288204. Table S1 presents the details of data collection and crystal structure determinations.

### 2.2 Pharmacology/Biology

#### 2.2.1 Cytotoxic Effects of the Ligands

Heterocyclic structures are crucial for the development of anticancer agents. Ring type and size, as well as the aliphatic and aromatic substituents on the scaffold, directly affect the physical, chemical, and biological properties of the structure.^20^ Effects of the synthesized ligands (**2**, **3a–c**) on cell viability were evaluated using various cancer cells and nontumorigenic embryonic kidney cells. All of the tested ligands (**2**, **3a–c**) exhibited considerable cytotoxic effects on cancer cells in a time- and concentration-dependent manner (see Figure S33). The most effective cytotoxic effects were observed after 72 h of incubation, and IC_50_ values were calculated at 72 h (see Table 1). With an IC_50_ of 3.9 ± 0.3 μM, ligand **3c** was more cytotoxic to the HEPG2 cells compared with the reference drug cisplatin. Ligand **3c** also affected the nontumorigenic HEK-293 cells less than the cancer cells, with an IC_50_ of 26.9 ± 1.6 μM.

**Table 1:** IC_50_ (μM) Values of **2**, **3a**–**c**, **4a**–**d** and Cisplatin in HEPG2, MCF-7, PC-3, and HEK-293 Cells at 72 h

compound	HEPG2 (hepatocellular carcinoma)	MCF-7 (human breast cancer)	PC-3 (human prostate cancer)	HEK-293 (human embryonic kidney)
**2**	226.5 ± 1.5	100.4 ± 2.4	58.2 ± 3.2	127.2 ± 2.8
**3a**	221.2 ± 0.8	156.6 ± 1.3	189.6 ± 0.9	116.8 ± 2.2
**3b**	42.95 ± 1.8	52.6 ± 3.1	58.1 ± 3.3	61.9 ± 0.6
**3c**	3.9 ± 0.3	27.2 ± 2.4	58.4 ± 2.0	26.9 ± 1.6
**4a**	552.78 ± 3.7	100.71 ± 2.6	228.6 ± 2.2	125.1 ± 3.2
**4b**	60.54 ± 2.4	46.69 ± 2.8	55.1 ± 1.5	52.8 ± 1.0
**4c**	41.95 ± 1.2	47.07 ± 3.1	46.4 ± 1.7	50.9 ± 0.8
**4d**	0.19 ± 0.4	0.54 ± 0.6	54.7 ± 3.0	22.4 ± 2.5
cisplatin	22.2 ± 0.5	20.8 ± 2.3	18.5 ± 0.7	10.7 ± 0.8

*N*-Alkylated indole-based compounds exhibit cancer cytotoxicity.^21^ In 2003, Nguyen et al. reported that the compound *N*-(3,4-dichlorobenzyl)-1*H*-indole-2,3-dione, an *N*-alkylisate derivative, induced apoptosis in human cancer cell lines but not in normal cells.^22^ In 2007, structure–activity relationship (SAR) studies by Vine and colleagues demonstrated that the addition of *N*-substituted isatins, an aromatic ring with one or three carbon atom linkers at N1, increased the cytotoxicity of allyl, 2′-methoxyethyl, and 3′-methylbutyl. Moreover, electron-withdrawing groups substituted at the meta- or para-position of the phenyl ring were preferred to the ortho orientation. A total of 9 of the 24 compounds screened demonstrated higher selectivity against leukemia and lymphoma cell lines and exhibited submicromolar half-maximal inhibitory concentration (IC_50_) values.^23^

#### 2.2.2 Cytotoxic Activities of the Complexes

**Figure 2:**
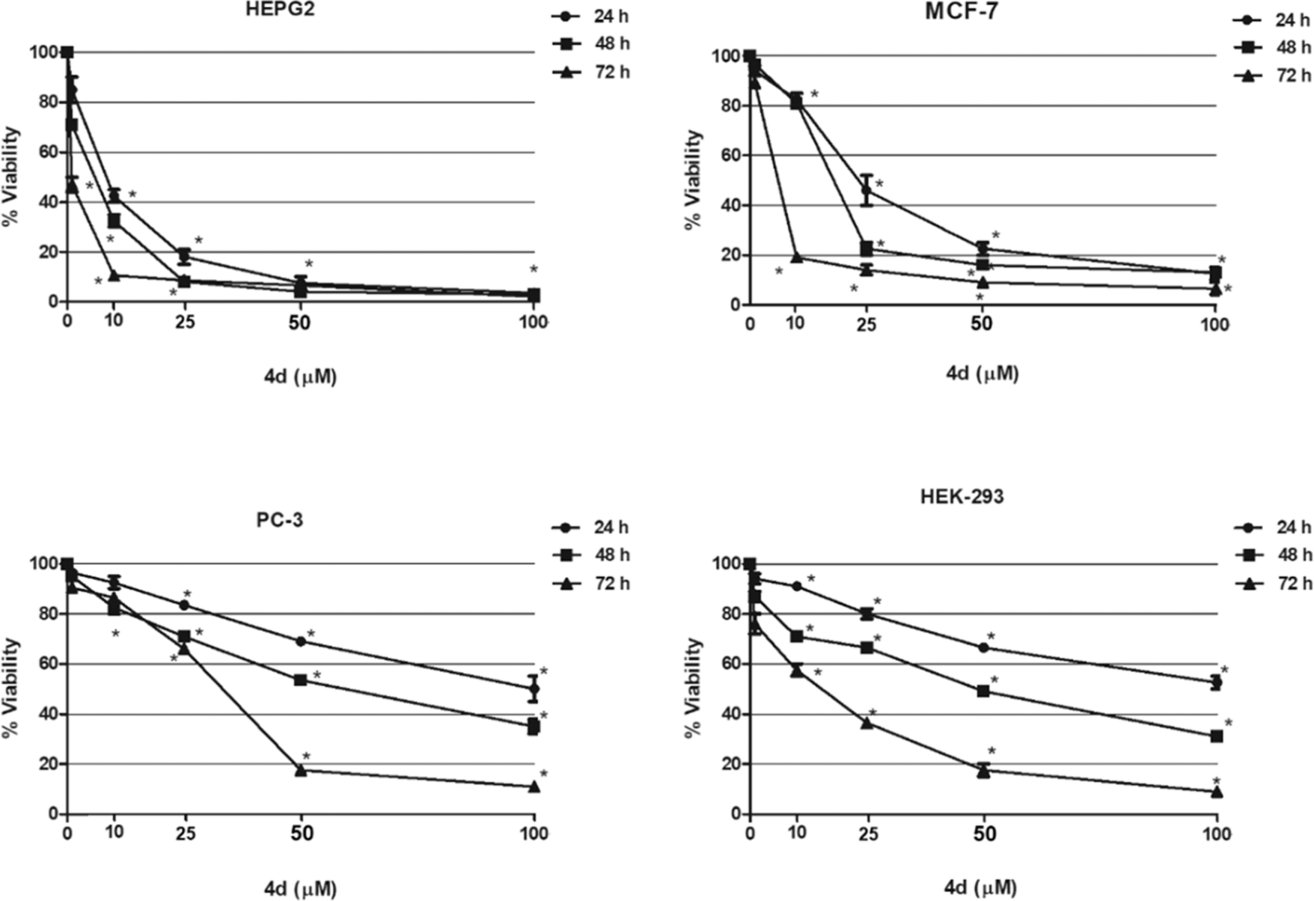
Cell viability results for complex **4d** in the cell lines HEPG2, MCF-7, PC-3, and HEK-293 after 24, 48, and 72 h of exposure.

Effects of the synthesized complexes (**4a**–**d**) on cell viability were evaluated using various cancer and nontumorigenic embryonic kidney cells. All of the tested complexes (**4a**–**d**) exhibited notable cytotoxic effects on cancer cells in a time- and concentration-dependent manner (see Figure 2). The results for the other complexes (**4a**–**c**) are provided in the Supporting Information (see Figure S34). The most effective cytotoxic effects were observed after 72 h of incubation, and the IC_50_ values were calculated at 72 h (see Table 1). Compared with the reference drug cisplatin, **4d** was more cytotoxic to HEPG2 and MCF-7 cells with IC_50_ values of 0.19 ± 0.4 and 0.54 ± 0.6 μM, respectively. **4d** also affected nontumorigenic HEK-293 cells less than cancer cells, with an IC_50_ of 22.4 ± 2.5 μM.

#### 2.2.3 Apoptotic Effects of the Complexes

**Figure 3:**
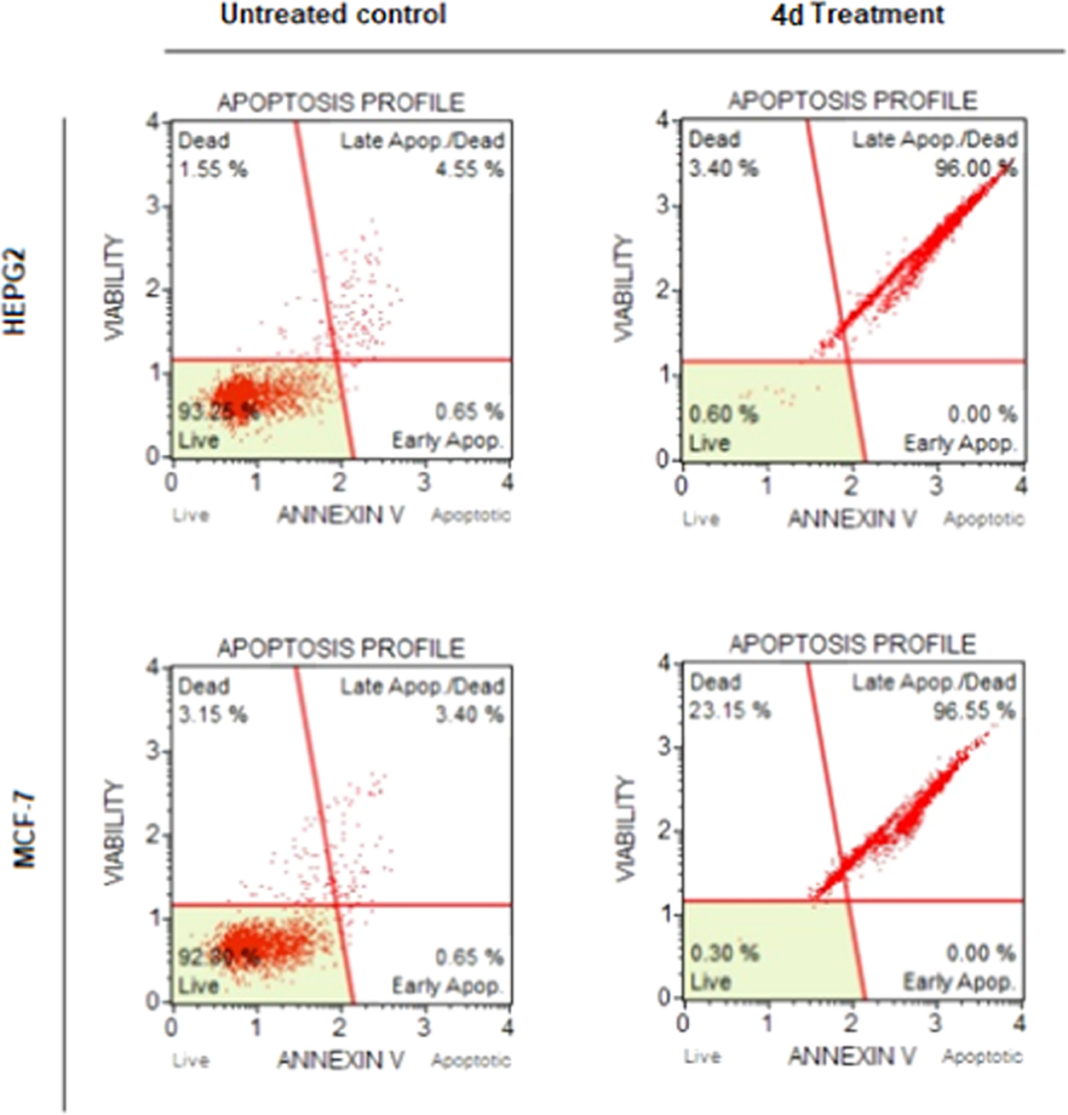
Apoptotic effect of complex **4d** on HEPG2 and MCF-7 cells at 72 h.

Complex **4d** was found to have considerable selective cytotoxic effects on HEPG2 and MCF-7 cancer cells. Annexin-V, known for its strong affinity for phosphatidylserine (PS), was employed to explore the apoptotic effects of **4d**. Fluorescein isothiocyanate (FITC) Annexin-V staining proved effective in identifying apoptosis at both early and late stages because PS externalization occurred during the early stages of apoptosis. HEPG2 and MCF-7 cancer cells were exposed to IC_50_ values of **4d** for 72 h, followed by flow cytometric analysis. Treatment of the cells with **4d** induced a strong apoptotic effect on them (see Figure 3). Treatment of HEPG2 cells with **4d** resulted in 96% apoptotic cells compared with the untreated control group (*p* < 0.05). A similar apoptotic effect (96.55% apoptotic cells) in MCF-7 cells was also observed after treatment with **4d** (*p* < 0.05).

**Figure 4:**
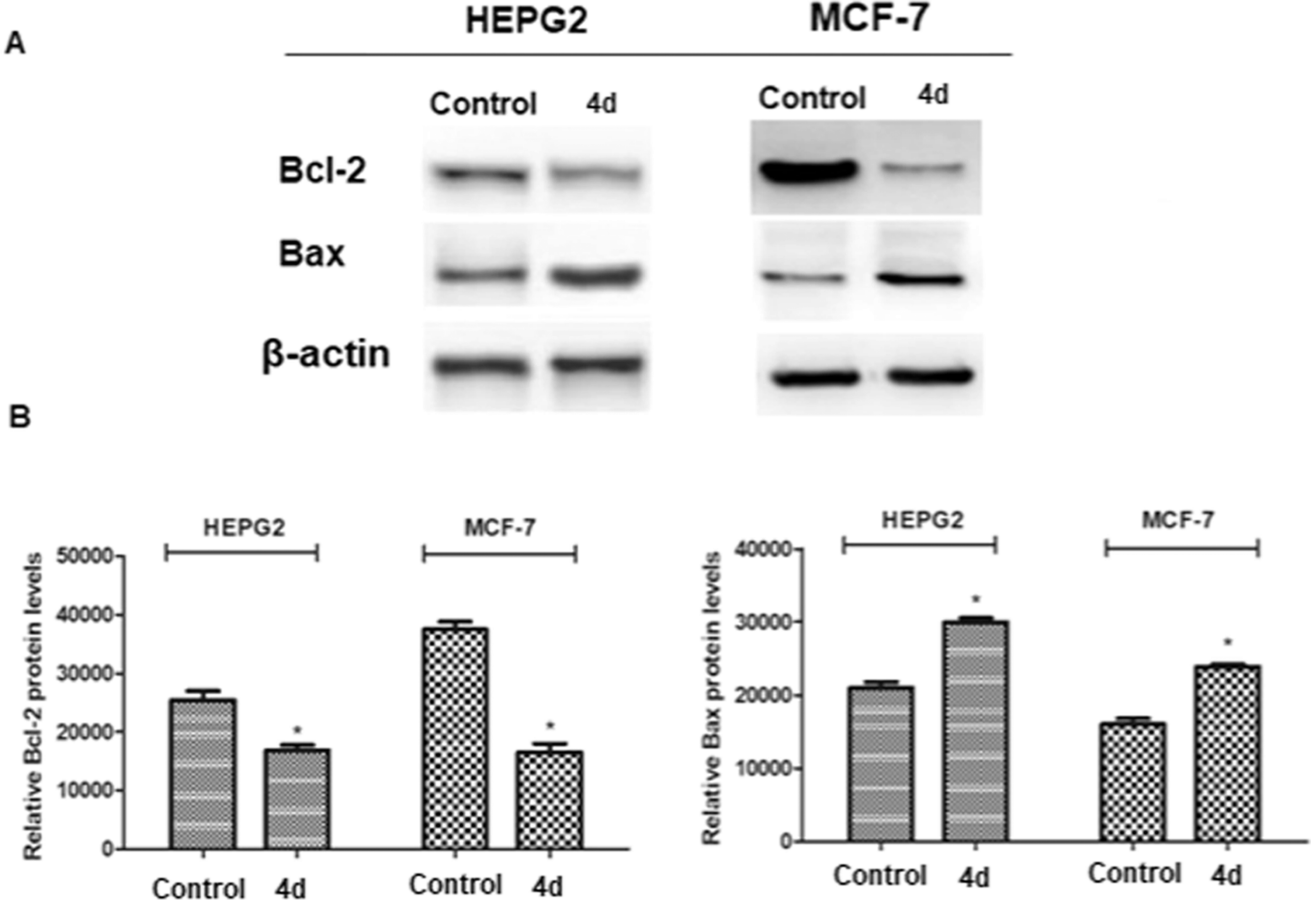
(A) Effect of complex **4d** on expression levels of proteins Bcl-2 and Bax. (B) Expression levels of Bcl-2 and Bax were quantified using the software ImageJ (*p < 0.05).

Levels of the apoptosis-related proteins Bcl-2 and Bax were evaluated via Western blot analysis to confirm the induction of apoptotic cell death by **4d** in HEPG2 and MCF-7 cells. Treatment with the IC_50_ value of **4d** resulted in a reduction in the Bcl-2 protein level and induction in Bax protein levels (see Figure 4A). The reduction in Bcl-2 levels was 1.5- and 2.27-fold in HEPG2 and MCF-7 cells, respectively (see Figure 4B, *p < 0.05). Induction in Bax protein levels was 1.6- and 1.69-fold in HEPG2 and MCF-7 cells, respectively (see Figure 4B, *p < 0.05).

#### 2.2.4 Stability of the Complexes

**Figure 5:**
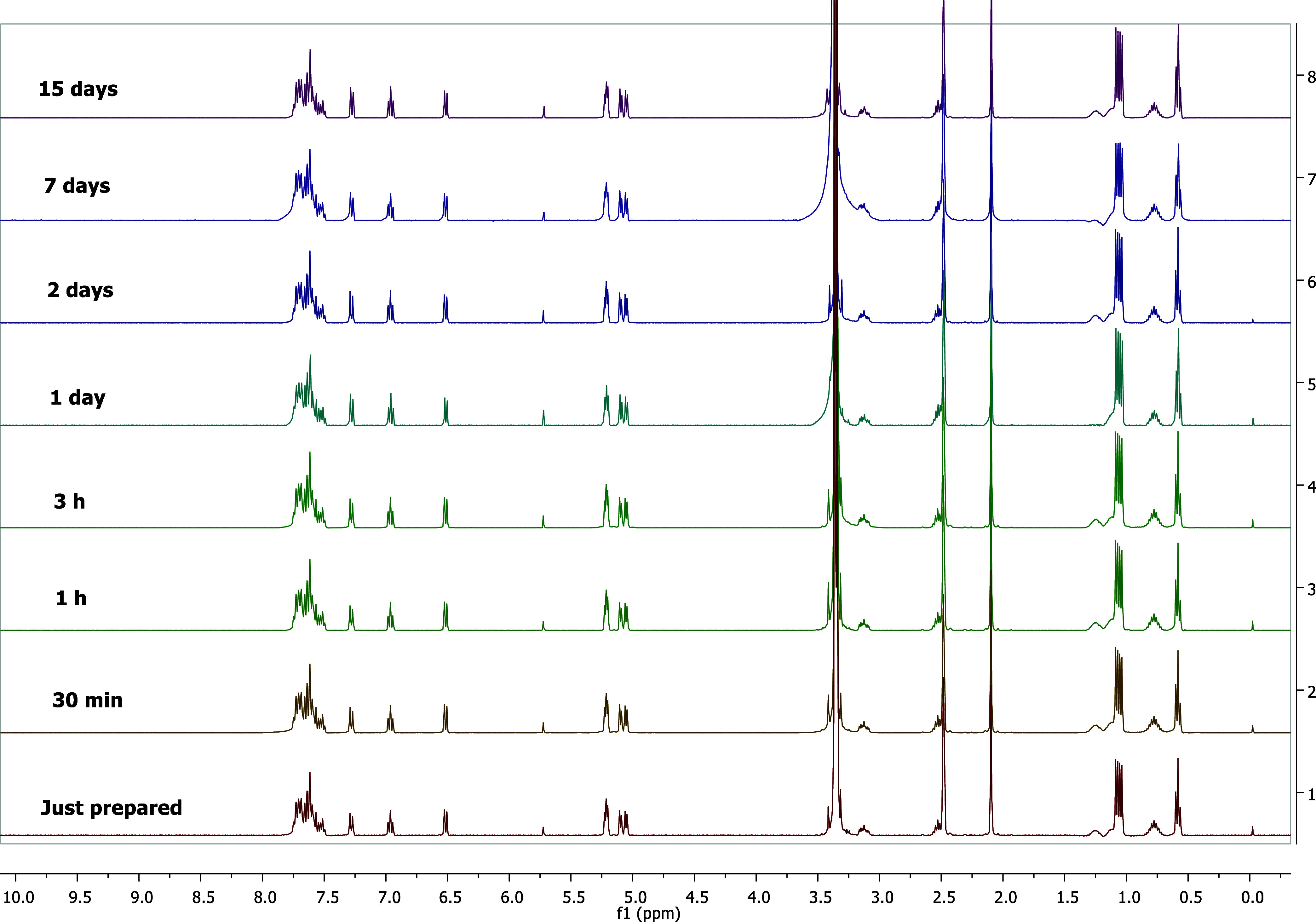
Stability of complex **4d** was tracked via ^1^H NMR spectroscopy in DMSO-*d*_6_ for 15 days.

For *in vitro* studies with metal complexes to be accurate and reliable, solvent and concentration parameters should be carefully evaluated, and the stability of the complex under certain conditions should be investigated. The stability of the complexes was evaluated by experimenting with different solvents, and NMR spectroscopies were employed to ensure the reliability of the results. Equilibrium studies on formation of Schiff base complexes cannot be conducted in aqueous solutions because of the nature of the compounds involved. These metal complexes and the ligands are insoluble in water.^24^ Therefore, stock solutions were prepared in DMSO to examine their biological properties because metal-based anticarcinogenic drugs have considerably low or no solubility in water or cell culture media.^25^ Thus, the chloride ion in the complex was replaced by molecules of cosolvents, such as DMSO. Aquation of the chloride ligand of a metal complex is important prior to binding to DNA. We investigated the time-dependent stability of **4b** (see Figure S35) and **4d** (see Figure 5) in a 100% DMSO-*d*_6_ solvent system via ^1^H NMR spectroscopy. Complexes **4b** and **4d** were rapidly hydrolyzed and attained equilibrium. They were stable in the solution as per the ^1^H NMR spectra for 15 days (complex **4d**) and 6 days (complex **4b**). In addition, we investigated the time-dependent stability of **4d** in the solvent system D_2_O/DMSO-*d*_6_ (20:80) via ^1^H NMR spectroscopy for 1 day (see Figure S36). The stability of complex **4d** in D_2_O/DMSO-*d*_6_ (20:80) gives a better understanding of the Cl/H_2_O exchange reaction.

#### 2.2.5 Interaction with FS–DNA

Metal complexes can interact with DNA via irreversible covalent (exchange a halogen) and reversible noncovalent (intercalation, electrostatic forces, or major/minor groove binding) interactions. Electronic absorption spectroscopy, called ultraviolet–visible (UV–vis) spectroscopy, is employed to investigate the binding properties between DNA and a complex. UV–vis analyses were performed to investigate the interactions of FS–DNA with **4d**. 0.1 mM FS–DNA was made to interact with **4d** (3–13 μM) in 20 mM Tris-HCl/NaCl (pH: 7.0). The interaction enhanced with increasing complex concentration with changes in absorption values. The measurement results of the maximum absorbance of the nucleic base chromophores of FS–DNA at 260 nm evidenced DNA insertion and were associated with noncovalent intercalation between DNA and the complex.^26^ The *K*_b_ of **4d** was (7.5 ± 0.1) × 10^5^ M^–1^ and is included in the Supporting Information (see Figure S37).

The results showed that **4d**, one of the Schiff base derivatives, binds in a tight and selective manner to DNA and is a cytotoxic agent in cancer cell lines HEPG2 and MCF-7. Our group reported synthesis of new mono- and bimetallic Ru(II)– and Ir(III)–arene complexes with various aromatic and aliphatic groups. These complexes, based on different ligands, exhibited less cytotoxicity on the cell lines Vero and HEPG2 versus cisplatin.^27^

#### 2.2.6 Lipophilicity

The experimental log *P* values of the studied compounds was determined via the direct extraction method. All compounds (**4a**–**d**) exhibited moderate lipophilicity with log* P* values in the range of 0.15–0.55 (see Table 2). This means that their solubility (**4b–d**), except **4a**, in *n*-octanol (lipophilic phase) is 2–3-fold higher than that in water (hydrophilic phase). The log* P* value of **4a** was lower than that of **4b**–**d** (log* P* = −0.017). In 2003, Domańska et al. reported that the octanol/water partition coefficient increases (i.e., it becomes less negative) as the number of carbon atoms in the alkyl group of the imidazole ring increases.^28^ CH_3_ groups on the benzene ring in **4c** increased its lipophilicity more than that of **4b**. The lipophilicity of ligands (**3a**–**d**) were determined by using ChemDraw 12.0, Molinspiration (www.molinspiration.com), and ALOGPS 2.1.^29^ The experimentally measured log *P* values for complexes (**4a**–**d**) do not differ as greatly as the calculated values for the free ligands (**3a**–**d**). In both cases, the order is unchanged. Furthermore, good correlation was found between the experimentally determined lipophilicity parameter and the computer predicted one calculated log *P*.

**Table 2:** Log *P* Values for the Ru(II) Complexes (**4a–d**) and Their Free Ligands

complex	log *P*	ligand	calculated log* P*
**4a**	–0.017	**3a**	3.24
**4b**	0.32	**3b**	4.74
**4c**	0.42	**3c**	5.95
**4d**	0.50	**3d**	7.15

The present results showed that the higher the lipophilicity, the higher the antitumor activity in complexes. The lipophilicity plays an important role in the anticancer activity of complexes of isatin Shiff bases and can be used for further studies of the complexes’ quantitative structure–activity relationships. From these observations, we can conclude that the higher the lipophilicity values, the higher the antitumor activity of the isatin Shiff base complexes synthesized.

## 3 Conclusions

We reported a series of Ru *p*-cymene complexes with Schiff bases and highly active **4d** cytotoxic agents for cancer cell lines HEPG2 and MCF-7. These complexes were characterized using spectroscopic methods. The effects of the synthesized complexes (**4a**–**d**) on cell viability were evaluated using different cancer cells and nontumorigenic HEK-293 cells. In addition to (3-(4,5-dimethylthiazol-2-yl)-2,5-diphenyltetrazolium bromide) (MTT) analyses, complex stability, DNA interaction, flow cytometry, and Western blotting studies were conducted. The aryl ligand types and *N*-alkylated ligand types on the Ru(II)–arene complexes may have resulted in direct mechanisms of action and caused differences in their cytotoxic activity studies. Consequently, the effects of the newly synthesized Ru complexes were observed depending on their dose on different cancer cell lines via MTT analysis. Among the complexes (**4a**–**d**), the highest cytotoxic activity was observed for **4d**, which was stable in the DMSO-*d*_6_ solvent system for 15 days. The stability of complex **4d** in D_2_O/DMSO-*d*_6_ (20:80) provided a better understanding of the Cl/H_2_O exchange reaction. We examined the interaction of **4d**, which showed effective cytotoxicity in the cell line HEPG2, with FS–DNA. The reactivity of isatin Schiff bases containing aryl and alkyl groups and their ruthenium(II) arene complexes was examined; we observed that addition of a secondary aryl group and *N*-alkyl-substituted isatins increased the reactivity and stability and resulted in an increase in cytotoxicity. The log*P* values measured by the direct shake-flask method range from −0.017 to 0.50. Studies on the lipophilicity of the complexes found that **4b**–**d** complexes have the best solubility in the *n*-octanol phase due to the addition of a secondary aryl group and *N*-alkyl substituted isatins. In general, cytotoxicity against cancerous cell lines appears to be more structure-dependent and more lipophilicity-dependent. We synthesized a therapeutic polyaromatic complex with high selectivity, i.e., **4d**, which will target only the cause of apoptosis of cancer cells.

## 4 Experimental Section

### 4.1 Chemistry

#### 4.1.1 General Information

Experimental procedures were realized with “Schlenk techniques” and “vacuum line techniques” for reactions that involved air-sensitive complexes under an argon atmosphere. The glass equipment was heated under a vacuum to remove oxygen and moisture, and then they were filled with argon. All chemicals and reagents were purchased from Merck, UPARC, and Alfa Aesar and used as received. ^1^H, ^13^C, ^19^F, and ^31^P NMR analyses were conducted on liquid Varian AS 400 MHz spectrometers. The J values are given in Hz. Single-crystal X-ray diffraction analysis was conducted at room temperature (RT) on a STOE IPDS II diffractometer using graphite-monochromated Mo Kα radiation by applying the ω-scan method. The melting points were measured on a Gallenkamp electrothermal melting point apparatus without correction. The FTIR spectra were recorded on PerkinElmer Spectrum 100 series equipment. Ligands **2** and **3a** were synthesized as per the literature.^16,30^ [RuCl_2_(*p*-cymene)]_2_ was prepared as per the method reported by Bennett and Smith via the reaction of ruthenium(III) chloride with α-terpinene.^31^

#### 4.1.2 General Procedure for Synthesis of **2**

Isatin **1** (3.00 g, 20.30 mmol), DMF (30 mL), and K_2_CO_3_ (2.81 g, 20.30 mmol) were charged in a balloon. 1-Bromobutane (2.78 g, 20.30 mmol) was dropwise added to the darkening red solution after 1 h. The reaction mixture was then refluxed and stirred for 2 h at 70 °C. After cooling, the mixture was poured into ice water, extracted with DCM (100 mL) three times, and purified on a silica gel column using DCM. (Color: reddish oil, 3.06 g, 74% yield). ^1^H NMR 400 MHz, CDCl_3_: δ 7.56 (t, *J* = 8.0 Hz, 2H, Ar-*H*), 7.09 (t, *J* = 7.6 Hz, 1H, Ar-*H*), 6.88 (d, *J* = 7.6 Hz, 1H, Ar-*H*), 3.70 (t, *J* = 7.2 Hz, 2H, butyl-*CH*_2_), 1.66 (m, 2H, butyl-*CH*_2_), 1.39 (m, 2H, butyl-*CH*_2_), 0.95 (t, *J* = 7.6 Hz, 3H, butyl-*CH*_3_). ^13^C NMR (100 Hz, CDCl_3_): δ 183.6, 158.1, 151.0, 138.4, 125.3, 123.5, 117.4, 110.2, 39.9, 29.2, 20.0, 13.6. IR, υ_max_ (KBr): 3060 (Ar–CH), 1735 (C=O), 1605 (C=C), 1330 (C=C), 1132 (C–N).

#### 4.1.3 General Procedure for Synthesis of **3a**

Isatin **1** (1.00 g, 6.79 mmol) was dissolved in 10 mL of hot ethanol. Aniline (0.63 g, 6.76 mmol) was dissolved in 2.5 mL of ethanol and added to the reaction. The reaction mixture was refluxed at 50 °C for 6 h. The reaction was completed, and the ethanol was distilled. Yellow crystals were obtained via recrystallization from ethanol (mp 224 °C, color: yellow, 1.28 g, 85% yield). Elemental analysis: calcd (%) for C_14_H_10_N_2_O (MW: 222.25): C, 75.66; H, 4.54; N, 12.60; O, 7.20%. Found: C, 75.67; H, 4.50; N, 12.65; O, 7.18%. ^1^H NMR 400 MHz, CDCl_3_: δ 9.83 (s, 1H, N-*H*), 7.44 (m, 2H, Ar-*H*), 7.28 (m, 2H, Ar-*H*), 7.04 (m, 2H, ArN-*H*), 6.95 (dd, *J* = 8.0 Hz, *J* = 2 Hz, 1H, Ar-*H*), 6.73 (m, 1H, Ar-*H*), 6.65 (d, *J* = 7.6 Hz, 1H, Ar-*H*). ^13^C NMR (100 Hz, CDCl_3_): δ 166.5, 154.7, 150.0, 145.7, 134.3, 129.4, 126.3, 125.4, 122.7, 117.8, 116.1, 111.9. IR, υ_max_ (KBr): 3458 (Ar-NH), 3164 (Ar–CH), 1739 (C=O), 1652 (C=N), 1590 (C=C), 1202 (C–N).

#### 4.1.4 General Procedure for Synthesis of **3b**

*N*-Butylisatine **2** (0.35 g, 1.72 mmol) was dissolved in 5 mL of absolute hot ethanol. Aniline (0.16 g, 1.72 mmol) was dissolved in 2.5 mL of ethanol and added to the reaction. The reaction mixture was refluxed for 6 h at 50 °C. The reaction was completed, and the ethanol was distilled. Orange crystals were obtained via recrystallization from ethanol (mp 130 °C, color: orange, 65% yield). Elemental analysis: calcd (%) for C_18_H_18_N_2_O (MW: 278.36): C, 77.67; H, 6.52; N, 10.06; and O, 5.75%. Found: C, 77.65; H, 6.54; N, 10.05; O, 5.76%. ^1^H NMR (400 MHz, CDCl_3_): δ 7.41 (t, *J* = 8.0 Hz, 2H, Ar-*H*), δ 7.32 (m, 1H, Ar-*H*), δ 7.22 (t, *J* = 7.6 Hz, 1H, Ar-*H*), δ 6.99 (d, *J* = 1.2 Hz, 1H, Ar-*H*), δ 6.97 (d, *J* = 0.8 Hz, 1H, Ar-*H*), δ 6.85 (d, *J* = 8.0 Hz, 1H, Ar-*H*), δ 6.71 (m, 1H, Ar-*H*), δ 6.59 (m, 1H, Ar-*H*), δ 3.78 (t, *J* = 7.2 Hz, 2H, butyl-*CH*_2_), δ 1.70 (m, 2H, butyl-*CH*_2_), δ 1.43 (m, 2H, butyl-*CH*_2_), δ 0.98 (t, *J* = 7.4 Hz, 3H, butyl-*CH*_3_). ^13^C NMR (100 Hz, CDCl_3_): δ 163.1, 154.4, 150.3, 147.5, 133.9, 129.3, 126.2, 125.1, 122.3, 117.7, 115.7, 109.4, 39.9, 29.3, 20.1, 13.7. IR, υ_max_ (KBr): 3083 (Ar–CH), 1718 (C=O), 1646 (C=N), 1602 (C=C), 1099 (C–N).

#### 4.1.5 General Procedure for Synthesis of **3c**

*N*-Butylisatine **2** (0.50 g, 2.46 mmol) was dissolved in 5 mL of hot ethanol. 2,4,6-Trimethylaniline (0.33 g, 2.46 mmol) was dissolved in 2.5 mL of ethanol and added to the reaction. The reaction mixture was refluxed at 50 °C for 24 h. The reaction was completed, and the ethanol was distilled. Crystals were obtained via recrystallization from ethanol (mp 145 °C, color: orange, 0.68 g, 86% yield). Elemental analysis: calcd (%) for C_21_H_24_N_2_O (MW: 320.44): C, 78.71; H, 7.55; N, 8.74; and O, 4.99%. Found: C, 78.69; H, 7.58; N, 8.76; O, 4.97%. ^1^H NMR (400 MHz, CDCl_3_): δ 7.33 (m, 1H, Ar-*H*), 6.91 (s, 2H, Ar-*H*), 6.84 (d, *J* = 7.6 Hz, 1H, Ar-*H*), 6.74 (m, 1H, Ar-*H*), 6.40 (dd, *J* = 7.6 Hz, *J* = 0.4 Hz, 1H, Ar-*H*), 3.79 (t, *J* = 8.0 Hz, 2H, butyl-*CH*_2_), 2.32 (s, 3H, Ar–*CH*_3_), 1.99 (s, 6H, Ar–*CH*_3_), 1.72 (m, 2H, butyl–C*H*_2_), 1.44 (m, 2H, butyl–C*H*_2_), 0.99 (t, *J* = 7.4 Hz, 3H, butyl–C*H*_3_). ^13^C NMR (100 Hz, CDCl_3_): δ 162.89, 155.14, 145.80, 145.71, 133.95, 133.67, 129.01, 125.24, 124.04, 122.96, 116.63, 109.24, 40.01, 29.46, 20.82, 20.23, 17.79, 13.77. IR, υ_max_ (KBr): 3050 (Ar–CH), 1732 (C=O), 1665 (C=N), 1602 (C=C), 1360 (C–N).

#### 4.1.6 General Procedure for Synthesis of **3d**

Aniline (0.20 g, 2.14 mmol) and NEt_3_ (9.08 g, 12.84 mmol) were dissolved in 10 mL of toluene and heated to 90 °C. Then, 0.50 mL of TiCl_4_ was added into the solution, following which some white fog appeared. Then, *n*-butylisatine **2** (0.14 g, 0.71 mmol) was added to the mixture, after which the solution darkened. The reaction mixture was stirred for 2 h. We then attempted to separate the mixture via DCM column chromatography, but it could not be isolated.

#### 4.1.7 General Procedure for Synthesis of **4a**

Compound **3a** (0.075 g, 0.33 mmol) was taken into a Schlenk tube made of vacuum gas, followed by adding [RuCl_2_(*p*-cymene)]_2_ (0.5 equiv). The reaction mixture was refluxed in chloroform at RT for 6 h. Thereafter, KPF_6_ (1 equiv) in acetonitrile prepared separately was added. The final mixture was stirred at RT for 1 h. It was then filtered. The filtrate was crystallized in a DCM/diethyl ether system after vacuuming. (m.p.: 270 °C, color: orange, 86 mg, 40% yield). Elemental analysis: calcd (%) for C_24_H_24_N_2_OClF_6_PRu (MW: 637.95): C, 45.18; H, 3.79; N, 8.78; O, 2.51%. Found: C, 45.20; H, 3.80; N, 8.81; O, 2.58%. ^1^H NMR (400 MHz, DMSO-*d*_6_): δ 11.00 (s, 1H, N-*H*), δ 7.57 (t, *J* = 7.6 Hz, 1H, Ar-*H*), δ 7.46 (m, 1H, Ar-*H*), δ 7.07 (m, 5H, Ar-*H*), δ 6.97 (m, 2H, Ar-*H*), δ 6.89 (d, *J* = 8.0 Hz, 1H, *p*-cymene-*H*), δ 6.54 (d, *J* = 8.0 Hz, 2H, *p*-cymene-*H*), δ 6.47 (d, *J* = 8.0 Hz, 1H, *p*-cymene-*H*), δ 2.81 (m, 1H, *p*-cymene–C*H*), δ 2.23 (s, 3H, *p*-cymene–C*H*_3_), δ 1.15 (d, *J* = 8.0 Hz, 6H, *p*-cymene–CH(C*H*_3_)). ^13^C NMR (100 Hz, DMSO-*d*_6_): δ 184.82, 163.92, 150.98, 147.40, 145.74, 138.82, 134.99, 130.05, 129.24, 126.51, 125.12, 123.21, 122.16, 117.66, 116.25, 114.43, 112.64, 111.98, 33.43, 24.43, 21.01. ^19^F NMR (376.2 MHz, CDCl_3_): −69.06, −71.19 (d, *J*_F–F_ = 711.0 Hz, P*F*_6_). ^31^P NMR (161.8 MHz, DMSO-*d*_6_): –(131.01–157.47) (septed, *J*_P–P_ = 713.5 Hz, *P*F_6_). IR, υ_max_ (KBr): 3411 (Ar-NH), 3123 (Ar–CH), 1680 (C=O), 1637 (C=C), 1072 (C–N), 837 (C–H).

#### 4.1.8 General Procedure for Synthesis of **4b**

Compound **3b** (0.05 g, 0.18 mmol) was taken into a Schlenk tube made of vacuum gas, followed by adding [RuCl_2_(*p*-cymene)]_2_ (0.5 equiv). The reaction mixture was stirred in chloroform at RT for 6 h. Thereafter, KPF_6_ (1 equiv) in acetonitrile prepared separately was added. The final mixture was stirred at RT for 1 h. It was then filtered. The filtrate was separated via column chromatography and crystallized in a DCM/diethyl ether system. (mp 188 °C, color: orange, 54.7 mg, 44% yield). Elemental analysis: calcd (%) for C_28_H_32_N_2_OClF_6_PRu (MW: 694.06): C, 48.45; H, 4.65; N, 8.07; O, 2.30%. Found: C, 48.50; H, 4.62; N, 8.11; O, 2.32%. ^1^H NMR (400 MHz, CDCl_3_): δ 7.88 (s, 1H, Ar-*H*), δ 7.69 (s, 1H, Ar-*H*), δ 7.5 (t, *J* = 8.0 Hz, 2H, Ar-*H*), δ 7.43 (t, *J* = 8.0 Hz, 2H, Ar-*H*), δ 6.92 (d, *J* = 8.0 Hz, 1H, Ar-*H*), δ 6.88 (d, *J* = 7.6 Hz, 1H, Ar-*H*), δ 6.54 (d, *J* = 7.6 Hz, 1H, Ar-*H*), δ 6.02 (d, *J* = 6.0 Hz, 1H, *p*-cymene-*H*), δ 5.78 (d, *J* = 6.0 Hz, 1H, *p*-cymene-*H*), δ 5.58 (d, *J* = 6.0 Hz, 1H, *p*-cymene-*H*), δ 5.55 (d, *J* = 5.6 Hz, 1H, *p*-cymene-*H*), δ 3.82 (m, 2H, butyl–C*H*_2_), δ 2.46 (m, 1H, *p*-cymene–C*H*), δ 1.85 (s, 3H, *p*-cymene–C*H*_3_), δ 1.75 (m, 2H, butyl–C*H*_2_), δ 1.43 (m, 2H, butyl–C*H*_2_), δ 1.28 (d, *J* = 7.2 Hz, 3H, *p*-cymene–CH(C*H*_3_)), δ 1.20 (d, *J* = 7.2 Hz, 3H, *p*-cymene–CH(C*H*_3_)), δ 0.97 (t, *J* = 7.2 Hz, 3H, butyl–C*H*_3_). ^13^C NMR (100 Hz, CDCl_3_): δ 172.42, 163.76, 149.89, 148.01, 137.20, 130.27, 129.77, 127.04, 125.10, 120.25, 114.67, 112.15, 102.86, 97.18, 84.28, 83.93, 82.40, 81.72, 41.58, 30.71, 28.92, 22.06, 21.81, 19.97, 17.60, 13.47. ^19^F NMR (376.2 MHz, CDCl_3_): −71.81, −73.66 (d, *J*_F–F_ = 711.0 Hz, P*F*_6_). ^31^P NMR (161.8 MHz, CDCl_3_): 114.58–96.74 (pented, *J*_P–P_ = 713.5 Hz, *P*F_6_). IR, υ_max_ (KBr): 3108 (Ar–CH), 1667 (C=O), 1608 (C=C), 1145 (C–N), 838 (C–H).

#### 4.1.9 General Procedure for Synthesis of **4c**

Compound **3c** (0.10 g, 0.31 mmol) was taken into a Schlenk tube made of vacuum gas, followed by adding [RuCl_2_(*p*-cymene)]_2_ (0.5 equiv). The reaction mixture was refluxed in chloroform for 12 h at 70 °C. Thereafter, KPF_6_ (1 equiv) in acetonitrile prepared separately was added. The final mixture was stirred at RT for 1 h. It was then filtered. The filtrate was separated via column chromatography and crystallized in a DCM/diethyl ether system. (m.p.: 220 °C, color: orange, 103 mg, 45% yield). Elemental analysis: calcd (%) for C_31_H_38_N_2_OClF_6_PRu (MW: 736.14): C, 50.58; H, 5.20; N, 7.60; O, 2.17%. Found: C, 50.62; H, 5.23; N, 7.57; O, 2.25%. ^1^H NMR (400 MHz, CDCl_3_): δ 7.51 (t, *J* = 7.8 Hz, 1H, Ar-*H*), δ 7.17 (s, 1H, Ar-*H*), δ 7.07 (s, 1H, Ar-*H*), δ 7.0 (d, *J* = 8.4 Hz, 1H, Ar-*H*), δ 6.93 (t, *J* = 7.8 Hz, 1H, Ar-*H*), δ 6.33 (d, *J* = 7.2 Hz, 1H, Ar-*H*), δ 6.04 (d, *J* = 5.6 Hz, 1H, *p*-cymene-*H*), δ 5.86 (d, *J* = 5.6 Hz, 1H, *p*-cymene-*H*), δ 5.53 (d, *J* = 5.6 Hz, 1H, *p*-cymene-*H*), δ 5.39 (d, *J* = 5.6 Hz, 1H, *p*-cymene-*H*), δ 4.07 and 3.72 (m, 2H, butyl–C*H*_2_), δ 2.58 (m, 1H, *p*-cymene–C*H*), δ 2.42 (s, 3H, Ar–C*H*_3_), δ 2.28 (s, 3H, Ar–C*H*_3_), δ 2.18 (s, 3H, Ar–C*H*_3_), δ 1.83 (s, 3H, *p*-cymene–C*H*_3_), δ 1.70 (m, 2H, butyl–C*H*_2_), δ 1.40 (m, 2H, butyl–C*H*_2_), δ 1.32 (d, *J* = 6.8 Hz, 3H, *p*-cymene–CH(C*H*_3_)), δ 1.27 (d, *J* = 6.8 Hz, 3H, *p*-cymene–CH(C*H*_3_)), δ 0.95 (t, *J* = 7.2 Hz, 3H, butyl–C*H*_3_). ^13^C NMR (100 Hz, CDCl_3_): δ 171.9, 166.5, 149.5, 143.4, 139.2, 137.8, 130.6, 130.3, 129.4, 127.7, 126.2, 125.7, 114.7, 112.5, 104.3, 95.3, 84.8, 83.9, 83.5, 81.4, 41.7, 30.9, 29.0, 21.9, 21.9, 20.9, 19.9, 19.6, 17.8, 17.3, 13.4. ^19^F NMR (376.2 MHz, CDCl_3_): −71.93, −73.99 (d, *J*_F–F_ = 711.0 Hz, P*F*_6_). ^31^P NMR (161.8 MHz, CDCl_3_): 115.47–97.79 (pented, *J*_P–P_ = 713.5 Hz, *P*F_6_). IR, υ_max_ (KBr): 3036 (Ar–CH), 1655 (C=O), 1611 (C=C), 1230 (C–N), 836 (C–H).

#### 4.1.10 General Procedure for Synthesis of **4d**

Compound **3d** (0.30 g, 0.85 mmol) was taken into a Schlenk made of vacuum gas, followed by adding [RuCl_2_(*p*-cymene)]_2_ (0.5 equiv). The reaction mixture was stirred in DCM at RT for 6 h. Thereafter, KPF_6_ (1 equiv) in acetonitrile prepared separately was added. The final mixture was stirred at RT for 1 h. It was then filtered. The filtrate was separated via column chromatography, and red crystals were obtained in a DCM/diethyl ether system (mp 224 °C, color: orange, 0.4 g, 61% yield). Elemental analysis: calcd (%) for C_34_H_37_N_3_ClF_6_PRu (MW: 769.17): C, 53.10; H, 4.84; N, 10.92%. Found: C, 53.14; H, 4.87; N, 10.89%. ^1^H NMR (400 MHz, CDCl_3_): δ 7.85 (m, 1H, Ar-*H*), δ 7.76 (d, *J* = 8.0 Hz, 1H, Ar-*H*), δ 7.65 (m, 5H, Ar-*H*), δ 7.55 (t, *J* = 7.4 Hz, 1H, Ar-*H*), δ 7.44 (m, 3H, Ar-*H*), δ 6.79 (m, 2H, Ar-*H*), δ 6.56 (d, *J* = 8.0 Hz, 1H, Ar-*H*), δ 5.29 (t, *J* = 6.0 Hz, 1H, *p*-cymene-*H*), 5.18 (d, *J* = 6.0 Hz, 1H, *p*-cymene-*H*), δ 4.85 (d, *J* = 6.0 Hz, 1H, *p*-cymene-*H*), δ 4.82 (d, *J* = 6.0 Hz, 1H, *p*-cymene-*H*), δ 3.18 (m, 2H, butyl–C*H*_2_), δ 2.63 (m, 1H, *p*-cymene-*CH*), δ 2.07 (s, 3H, *p*-cymene-*CH*_3_), δ 1.28 (m, 2H, butyl-*CH*_2_), δ 1.15 (d, *J* = 6.8 Hz, 3H, *p*-cymene–CH(C*H*_3_)), δ 1.03 (d, *J* = 6.8 Hz, 3H, *p*-cymene–CH(C*H*_3_)), δ 0.85 (m, 2H, butyl–C*H*_2_), δ 0.65 (t, *J* = 7.4 Hz, 3H, butyl–C*H*_3_). ^13^C NMR (100 Hz, CDCl_3_): δ 168.4, 157.7, 153.4, 149.8, 147.7, 136.4, 130.2, 129.8, 129.5, 129.2, 123.3, 126.2, 123.2, 122.6, 121.7, 121.0, 115.0, 110.9, 108.8, 101.5, 86.8, 85.7, 85.2, 84.2, 43.5, 30.5, 29.0, 22.2, 21.4, 19.5, 17.9, 13.2. ^19^F NMR (376.2 MHz, CDCl_3_): −71.87, −73.59 (d, *J*_F–F_ = 711.0 Hz, P*F*_6_). ^31^P NMR (161.8 MHz, CDCl_3_): 114.30–96.65 (pented, *J*_P–P_ = 713.5 Hz, *P*F_6_). IR, υ_max_ (KBr): 3062 (Ar–CH), 1649 (C=N), 1649 (C=C), 1093 (C–N), 837 (C–H).

#### 4.1.11 XRD Analysis

Single-crystal X-ray diffraction data for **4d** were collected at RT on a STOE IPDS II diffractometer by using graphite-monochromated Mo Kα radiation by applying the ω-scan method. Data collection and cell refinement were performed using X-AREA, and data reduction was applied using X-RED32.^32^ The crystal structure was solved with the ShelXT solution program using dual methods and using Olex2 as the graphical interface.^19,33^ The model was refined using ShelXL via full matrix least-squares minimization on *F*^2^.^33^

### 4.2 Pharmacological/Biological Assays

#### 4.2.1 Cell Viability and Cytotoxicity

HEPG2 (ATCC-HB-8065), MCF-7 (ATCC-CRL-3435), PC-3 (ATCC-CRL-1435), and HEK-293 (ATCC-CRL-1573) cells were purchased from American Type Culture Collection (ATCC). Human cancer cells and embryonic kidney cells were maintained in Roswell Park Memorial Institute (RPMI) media supplemented with 10% fetal bovine serum and 1% penicillin–streptomycin solution in a 37 °C incubator with 5% CO_2_.

#### 4.2.2 MTT Cell Viability Assay

The cell viability was assessed by using an MTT assay. MTT was purchased from Sigma and prepared in phosphate-buffered saline (5 mM). Cells were seeded at a density of 10^4^ cells/well in 96-well cell culture plates and treated with increasing concentrations of **4a**–**d** derivatives for 24, 48, and 72 h. After incubation, 20 μL of MTT solution was added to each well and incubated at 37 °C for an additional 4h. Finally, the MTT solution was removed, and 200 μL of DMSO was added to each well. The absorbance was measured at 590 nm by using a spectrometer (Tecan). The IC_50_ values were calculated from the MTT viability data using the software CalcuSyn (Biosoft).

#### 4.2.3 Flow Cytometric Analysis of Apoptotic Cells

Apoptotic cells were analyzed using a flow cytometer (Muse Cell Analyzer). The Annexin-V/PI staining assay was performed to detect apoptotic cells using Muse Annexin-V and Dead Cell kit (Millipore, Billerica, MA, MCH100105) according to the manufacturer’s instructions. Briefly, the cells were seeded in a 6-well plate at a density of 4 × 10^5^ cells in 2 mL of cell culture medium. They were exposed to IC_50_ values of the derivatives and incubated for 72 h. The cells (500 μL) were treated with Muse Annexin-V & Dead Cell solution (500 μL) for 15 min at RT in the dark. Next, 400 μL of 1× binding buffer was added to each well, and flow cytometric analysis was performed for 100,000 cells.

#### 4.2.4 Western Blot Analysis

Total protein isolation was performed by using the M-PER Mammalian Protein Extraction Reagent (Thermo Fisher). The isolated protein concentrations were evaluated using the Bradford protein assay, and the proteins were equally separated on a SDS PAGE gel. The separated proteins were transferred to nitrocellulose membranes (Bio-Rad) and blocked using 5% nonfat dry milk (with 0.1% Tween 20). The membranes were incubated with primary antibodies (Bcl-2, Bax and β-actin) overnight at 4 °C and washed three times with TBST (Tris-buffered saline, 0.1% Tween 20). The membranes were then treated with secondary antibodies (1:1000 dilutions, SantaCruz) for 2 h at RT and washed several times with TBST (see Figure 4). Protein bands were visualized using UVP Imaging equipment, and the software ImageJ was used to quantify the protein bands.

#### 4.2.5 DNA Binding

DNA binding experiments were performed using 0.1 mM FS–DNA in Tris-HCl buffer (20 mM Tris-HCl/NaCl, pH 7.0) via UV spectroscopy of ruthenium complex **4d**. The Benesi–Hildebrand equation was used to calculate the *K*_b_. One has the following:
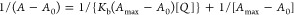
where *A*_0_ represents the absorption intensity of DNA at 260 nm in the absence of binding to the complex, *A*_max_ is the highest concentration of the DNA–metal complex combination, *A* is the concentration of DNA interacting with the metal complex, and [*Q*] is the concentration of the metal complex that provides binding. The *K*_b_ was graphically evaluated by plotting 1/[*A* – *A*_0_] versus 1/[*Q*].^34^

#### 4.2.6 Lipophilicity

1-Octanol/water partition coefficient (log *P*) indicated the lipophilicity of the molecules. Partition coefficients (*P*) between n-octanol and water phases of all synthesized ruthenium(II) complexes were determined using the extraction method. Test substances were prepared at a 1 mg/mL concentration. After applying the extraction method, both phases of the complex were evaporated via vacuum distillation and the amount of **4a**–**d** substance was calculated.

#### 4.2.7 Statistical Analysis

Statistical analysis was conducted using the software GraphPad Prism. The data were analyzed using a one-way analysis of variance test, followed by Dunnett’s test for multiple comparisons. *p* < 0.05 was accepted as statistically significant.

## Supporting Information Available

^1^H and ^13^C NMR spectra of **2**, **3a**, **3b**, **3c**, **4a**, **4b**, **4c**, and **4d**; ^19^F NMR spectrum of **4a**, **4b**, **4c**, and **4d**; ^31^P NMR spectrum of **4a**, **4b**, **4c**, and **4d**; FTIR spectrum of **3a**, **3b**, **3c**, **4a**, **4b**, **4c**, and **4d**; crystal data and structure refinement parameters for **4d**; pharmacological analysis results of **4d**; and UV–vis spectra of FS-DNA of **4d** (PDF)
