# Supplementary material for: Synthesis and Characterization of Piano-Stool Ruthenium(II)–Arene Complexes of Isatin Schiff Bases: Cytotoxicity and DNA Intercalation
Source: ACS Omega. 2024 Apr 17;9(17):19136–47. doi: 10.1021/acsomega.3c10265 (PMC11064044; doi:10.1021/acsomega.3c10265)
Supplement: Supplementary file 1 — ao3c10265_si_001.pdf [file ao3c10265_si_001.pdf]

Supporting Information

Synthesis and Characterisation of Piano-Stool Ruthenium (II)–Arene Complexes of Isatin Schiff Bases: Cytotoxicity and DNA Intercalation

Hande Karabıyık,[1] Aslıhan Karaer Tunçay,[2] Suleyman Ilhan,[3] Harika Atmaca,[3] Hayati Türkmen,[2*]

[1] Dokuz Eylül University, Faculty of Science, Department of Physics, Izmir, Turkey

[2]Department of Chemistry, Faculty of Science, Ege University, Bornova, 35100 Izmir, Turkey

[3]Department of Biology, Faculty of Engineering and Natural Sciences, Manisa Celal Bayar University, Manisa, Turkey

Contents

^{1}H and ^{13}C NMR spectra of **2, 3a, 3b, 3c, 4a, 4b, 4c, 4d**

^{19}F NMR spectra of **4a**, **4b, 4c, 4d**

1. ^{31}P NMR spectra of **4b, 4c, 4d**

FTIR spectra of **3a, 3b, 3c, 4a, 4b, 4c, 4d**

Crystal data and structure refinement parameters for **4d**

Cell viability results of the **2**, **3a**, **3b**, **3c**, **4a, 4b, 4c**

The stability of complexes **4b** and **4d**

FS-DNA structure analysis of **4d**

^{1}H-NMR spectra of 2

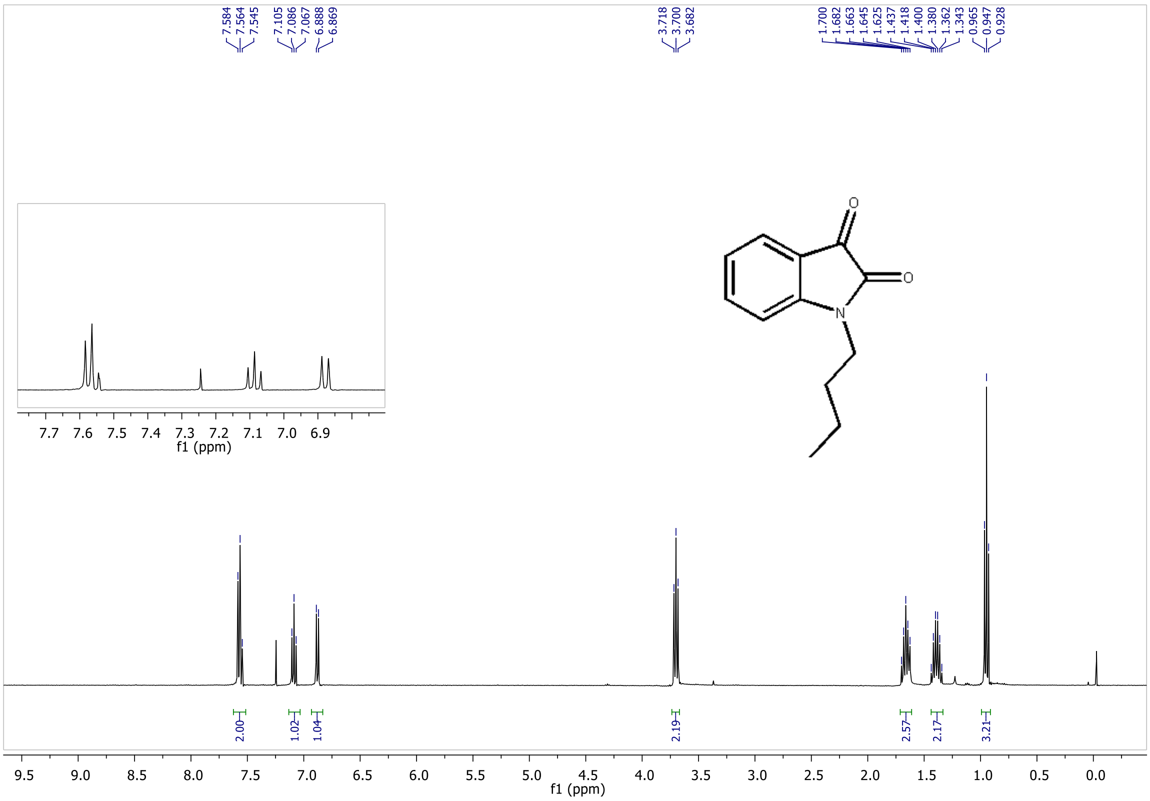

Figure S1. ^{1}H-NMR spectrum of **2** ($CDCl_3$).

^{13}C-NMR spectra of 2

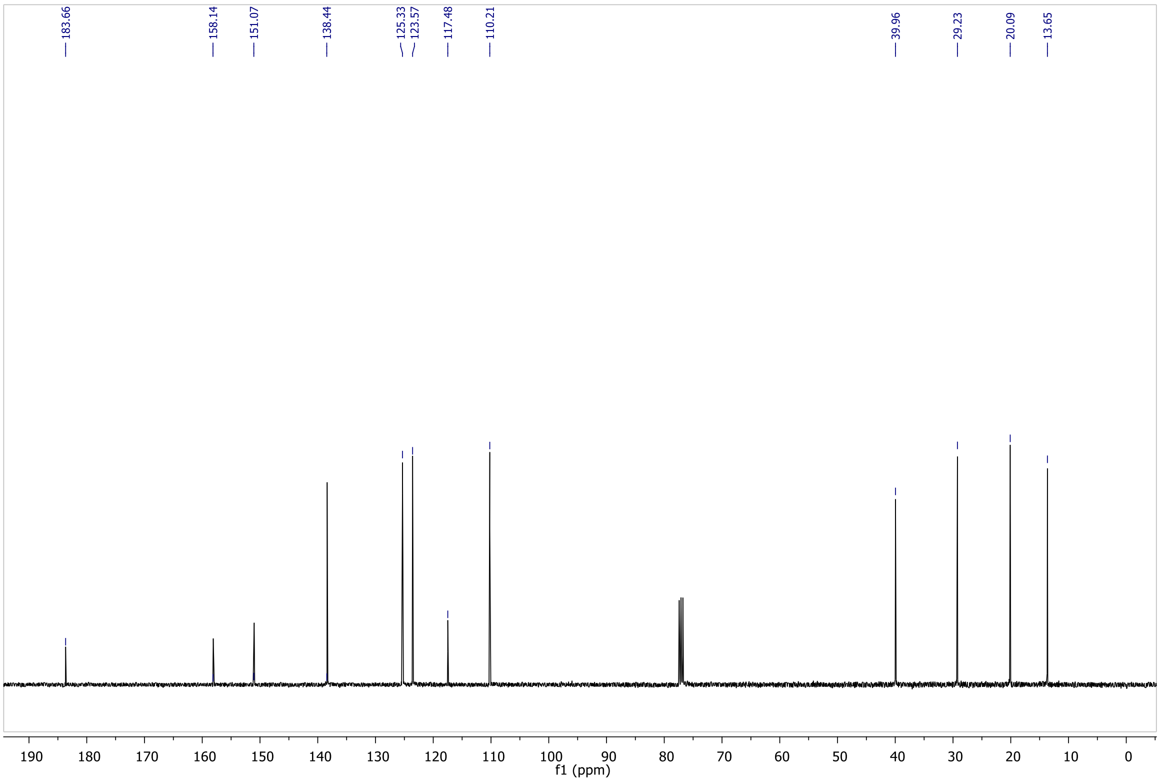

Figure S2. ^{13}C-NMR spectrum of **2** ($CDCl_3$).

^{1}H-NMR spectra of 3a

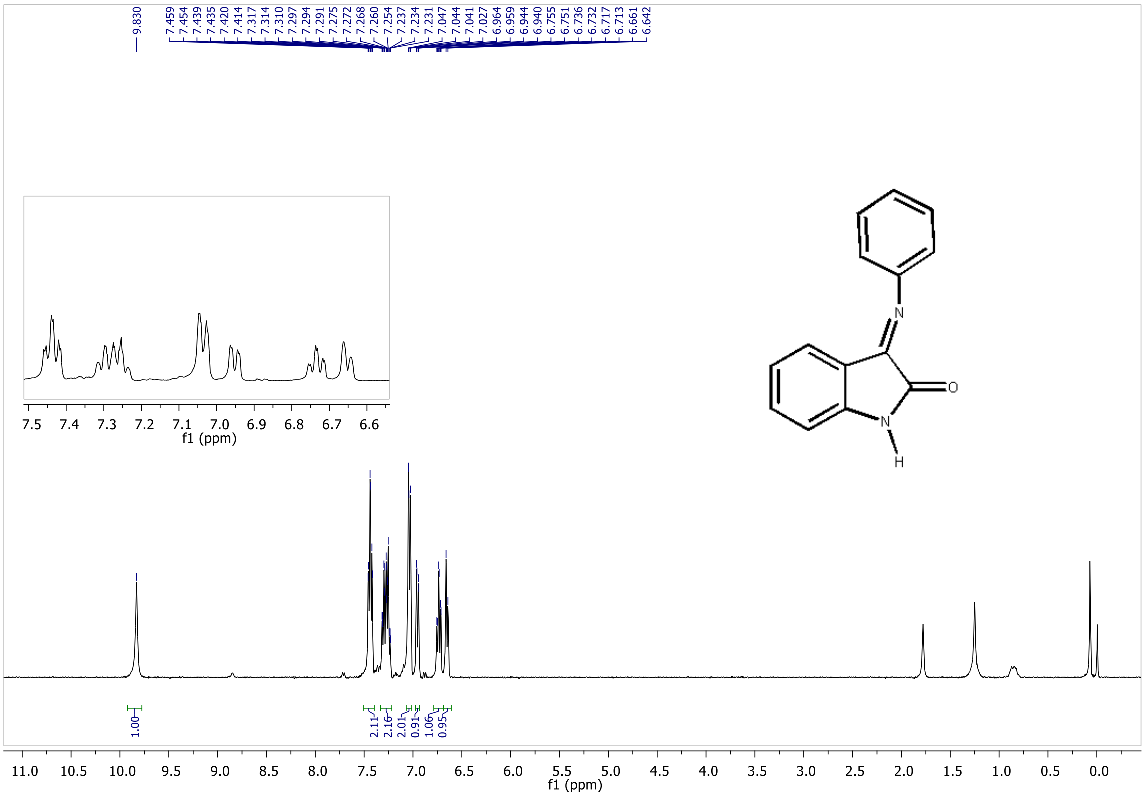

Figure S3. ^{1}H-NMR spectrum of **3a** ($CDCl_3$).

^{13}C-NMR spectra of 3a

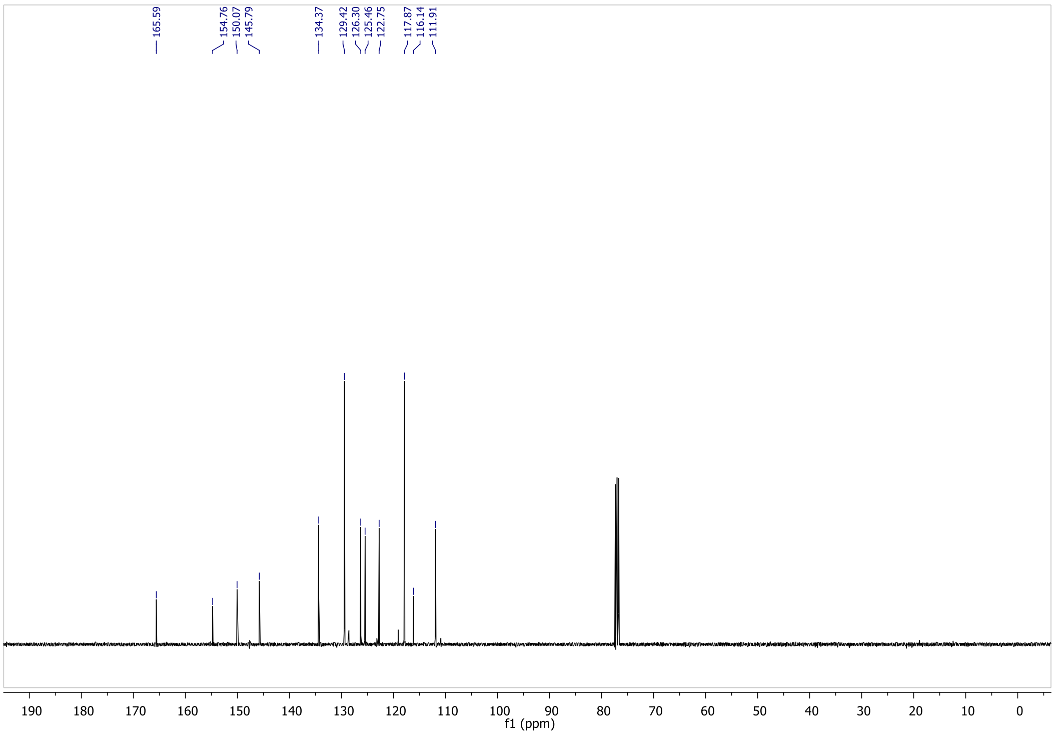

Figure S4. ^{13}C-NMR spectrum of **3a** ($CDCl_3$).

FTIR spectra of 3a

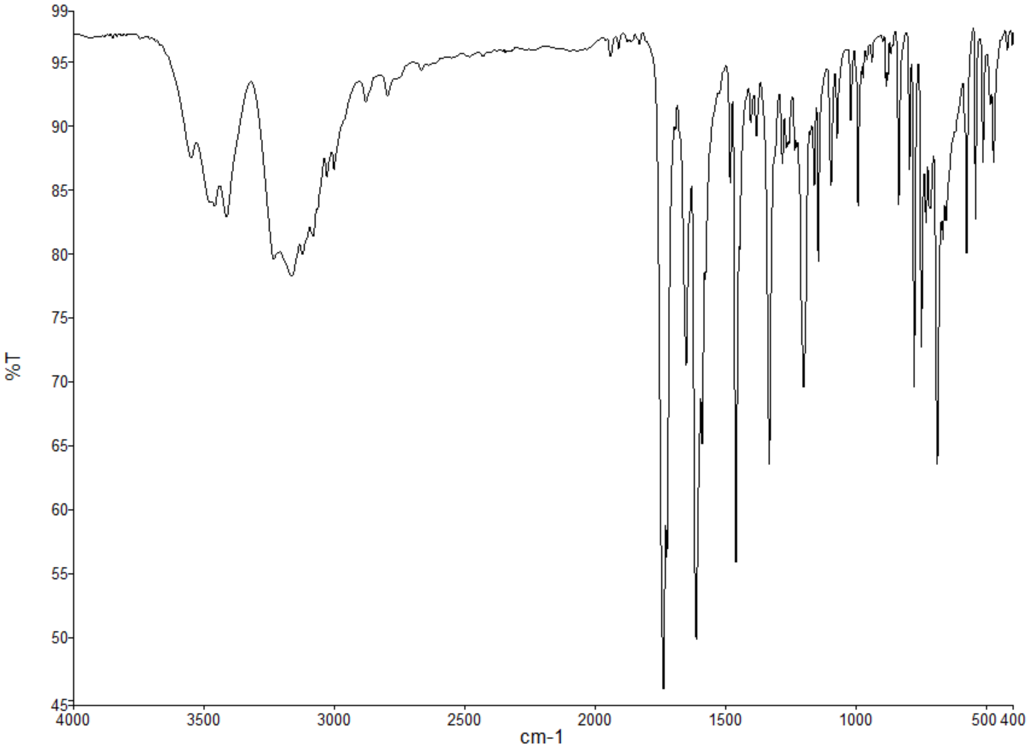

Figure S5. IR spectrum of **3a**.

^{1}H-NMR spectra of 3b

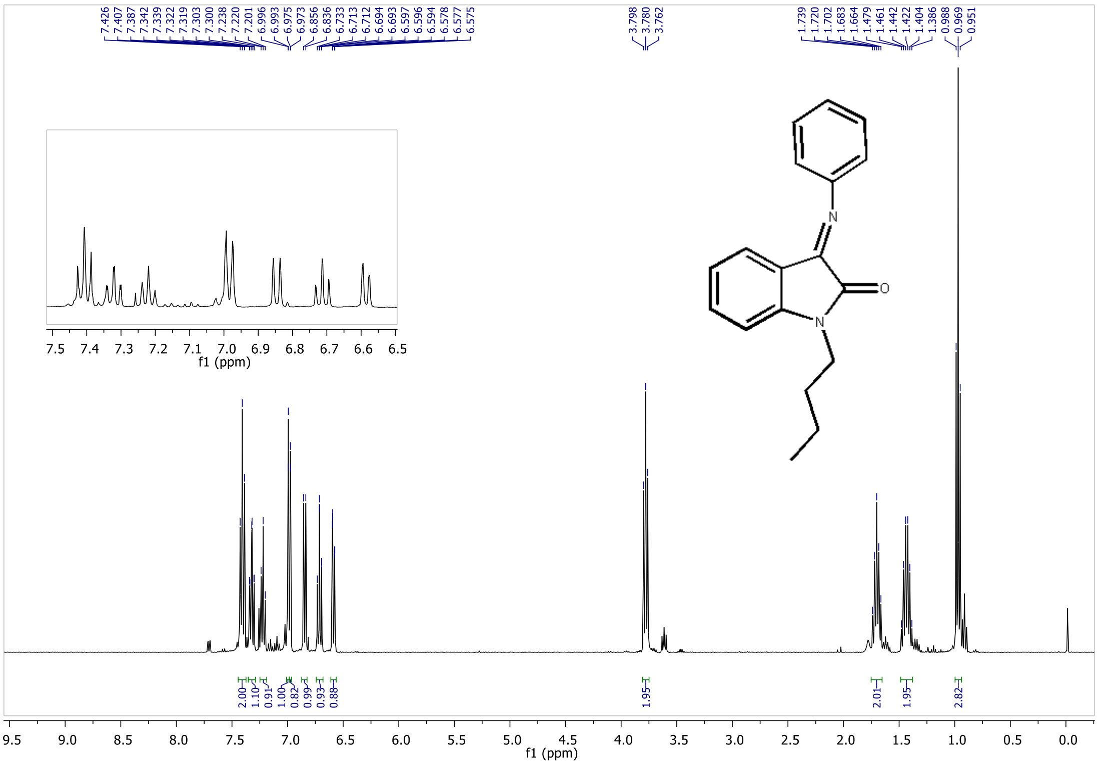

Figure S6. ^{1}H-NMR spectrum of **3b** ($CDCl_3$).

^{13}C-NMR spectra of 3b

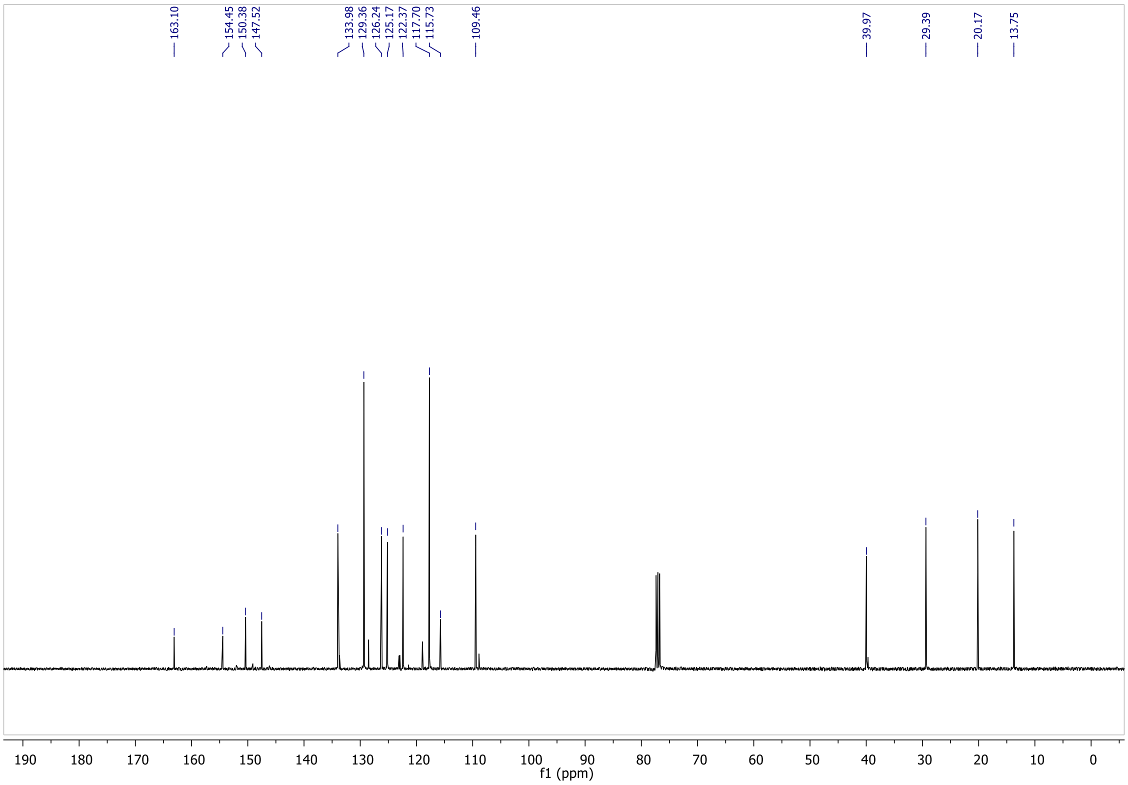

Figure S7. ^{13}C-NMR spectrum of **3b** ($CDCl_3$).

FTIR spectra of 3b

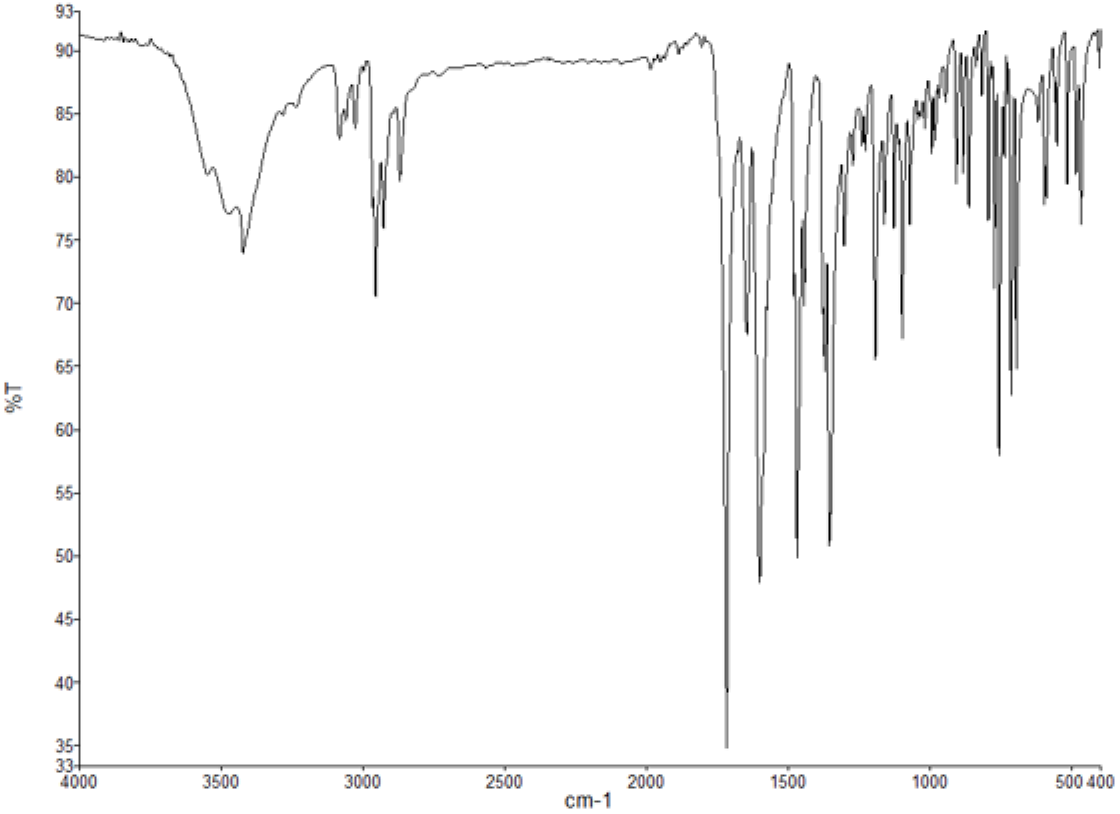

Figure S8. IR spectrum of **3b**.

^{1}H-NMR spectra of 3c

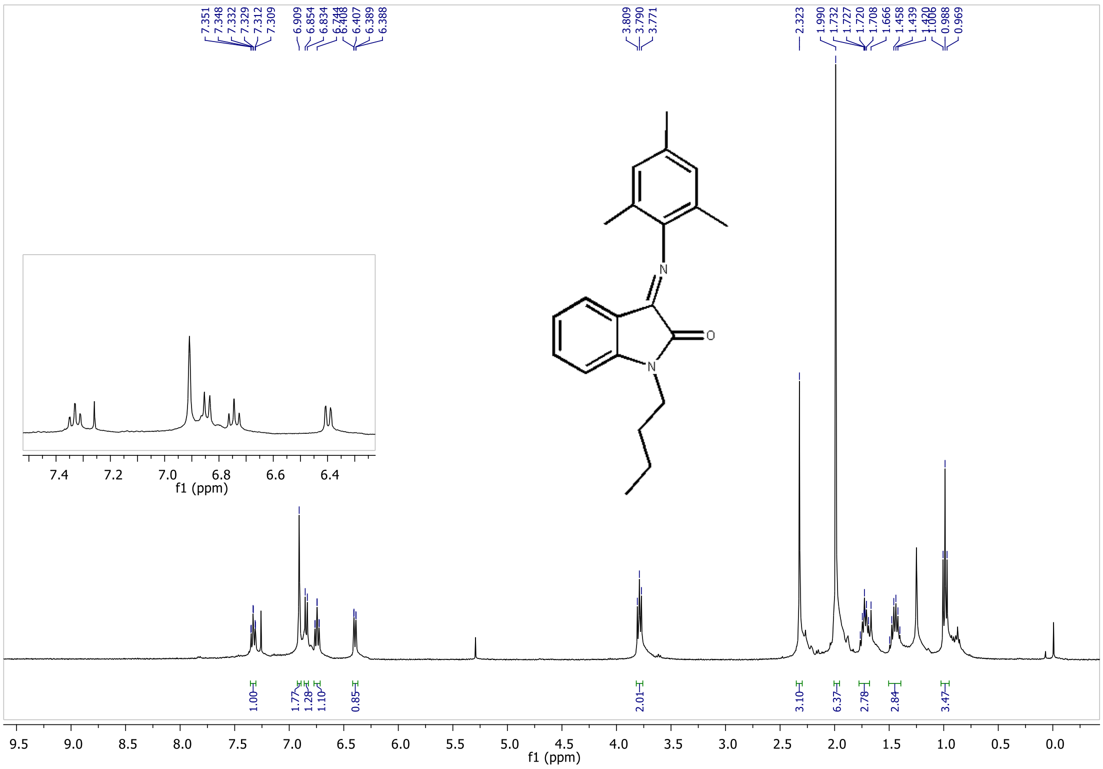

Figure S9. ^{1}H-NMR spectrum of **3c** ($CDCl_3$).

^{13}C-NMR spectra of 3c

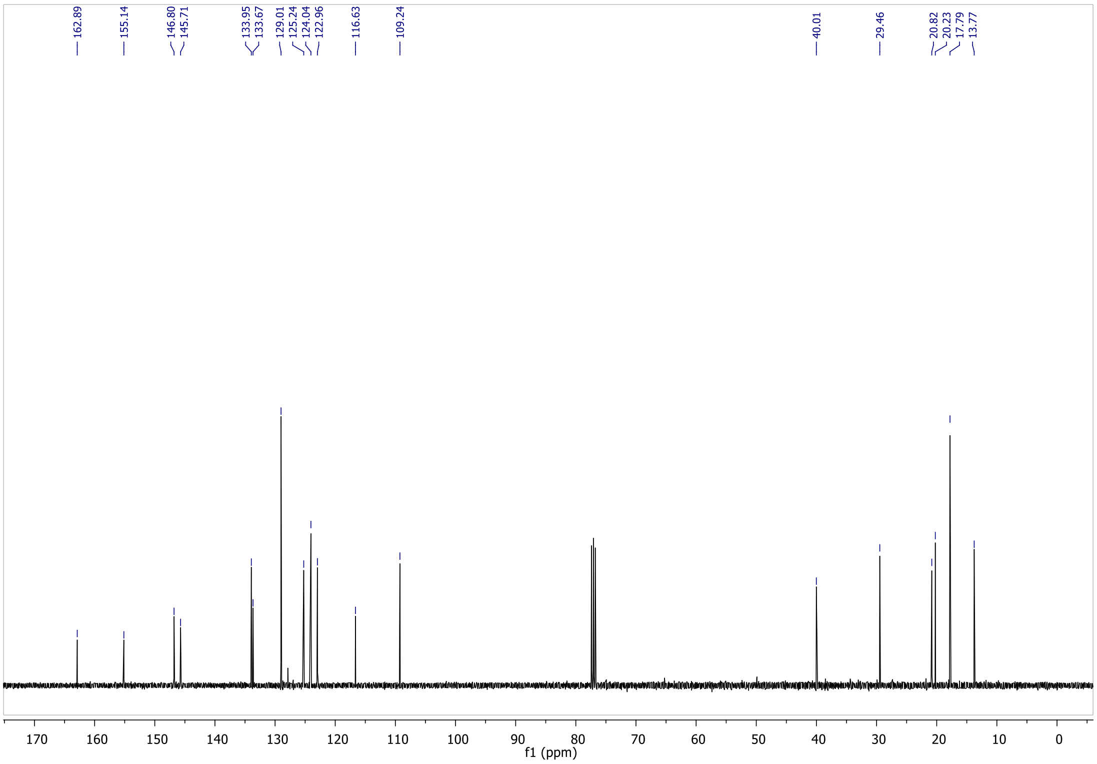

Figure S10. ^{13}C-NMR spectrum of **3c** ($CDCl_3$).

FTIR spectra of 3c

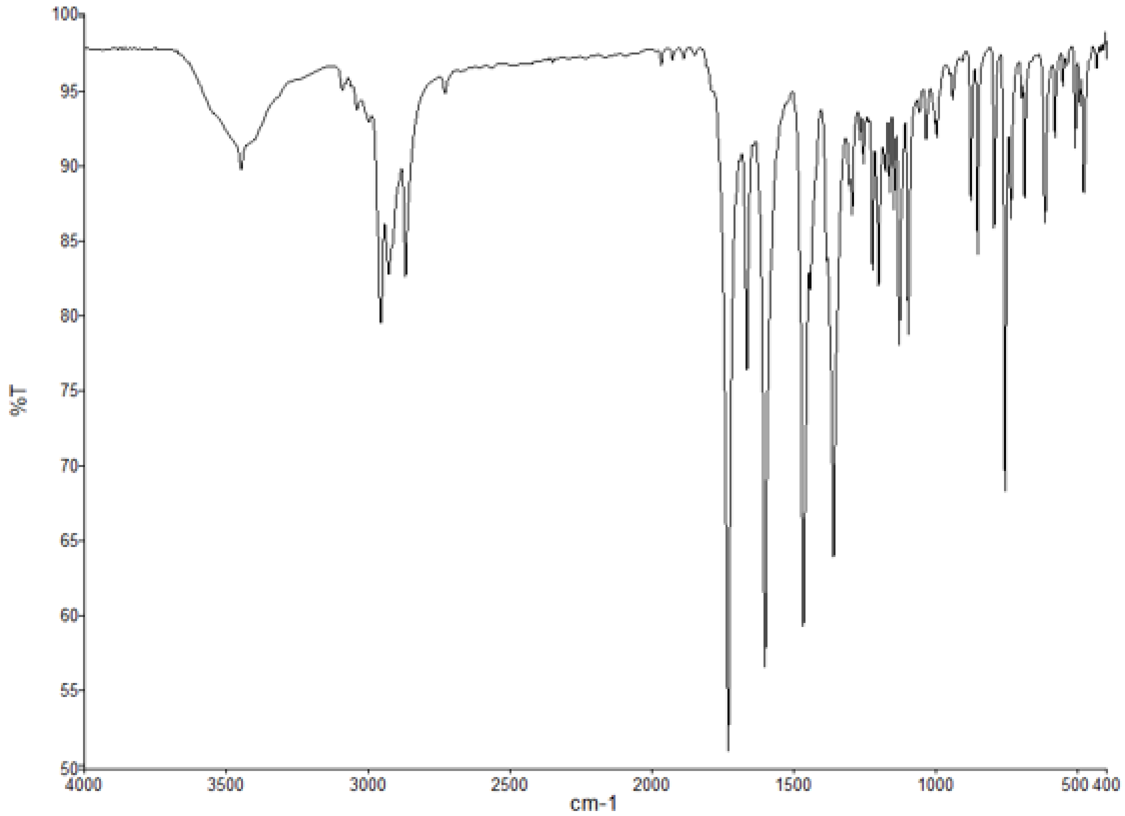

Figure S11. IR spectrum of **3c**.

^{1}H-NMR spectra of 4a

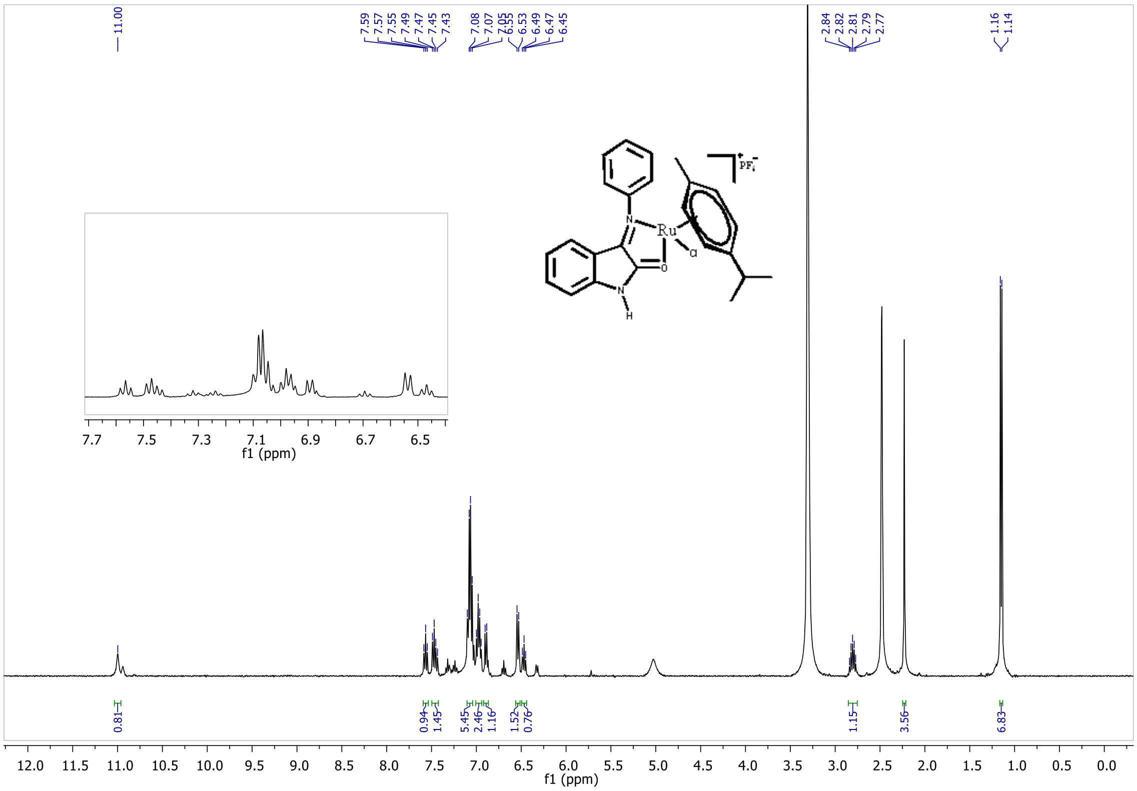

Figure S12. ^{1}H-NMR spectrum of **4a** (DMSO-d_6).

^{13}C-NMR spectra of 4a

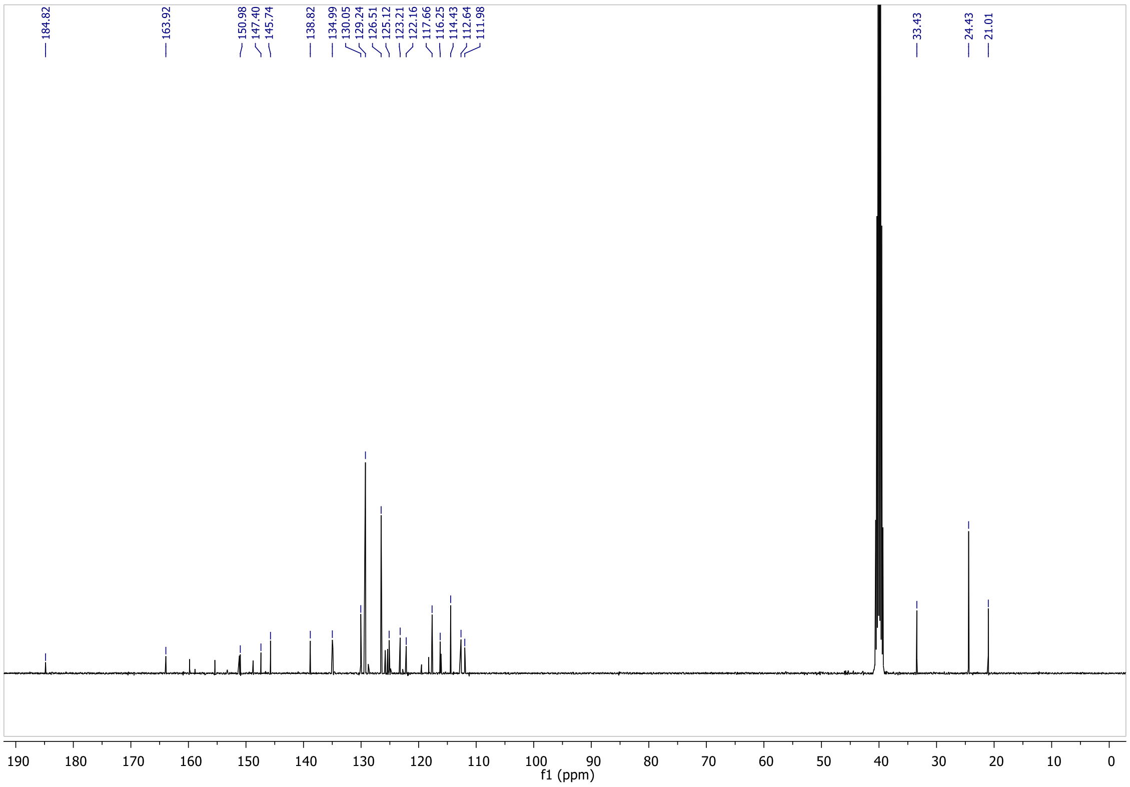

Figure S13. ^{13}C-NMR spectra of **4a** (DMSO-d_6).

FTIR spectra of 4a

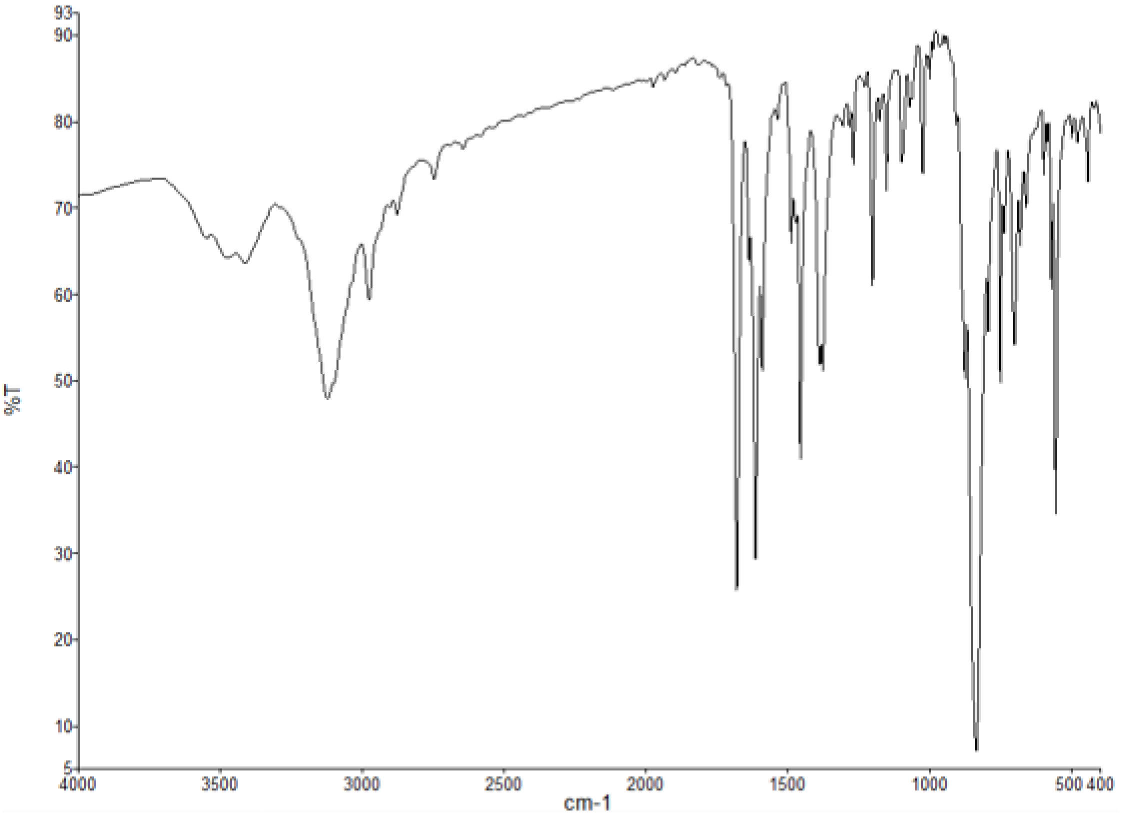

Figure S14. IR spectrum of **4a**.

^{19}F-NMR spectra of 4a

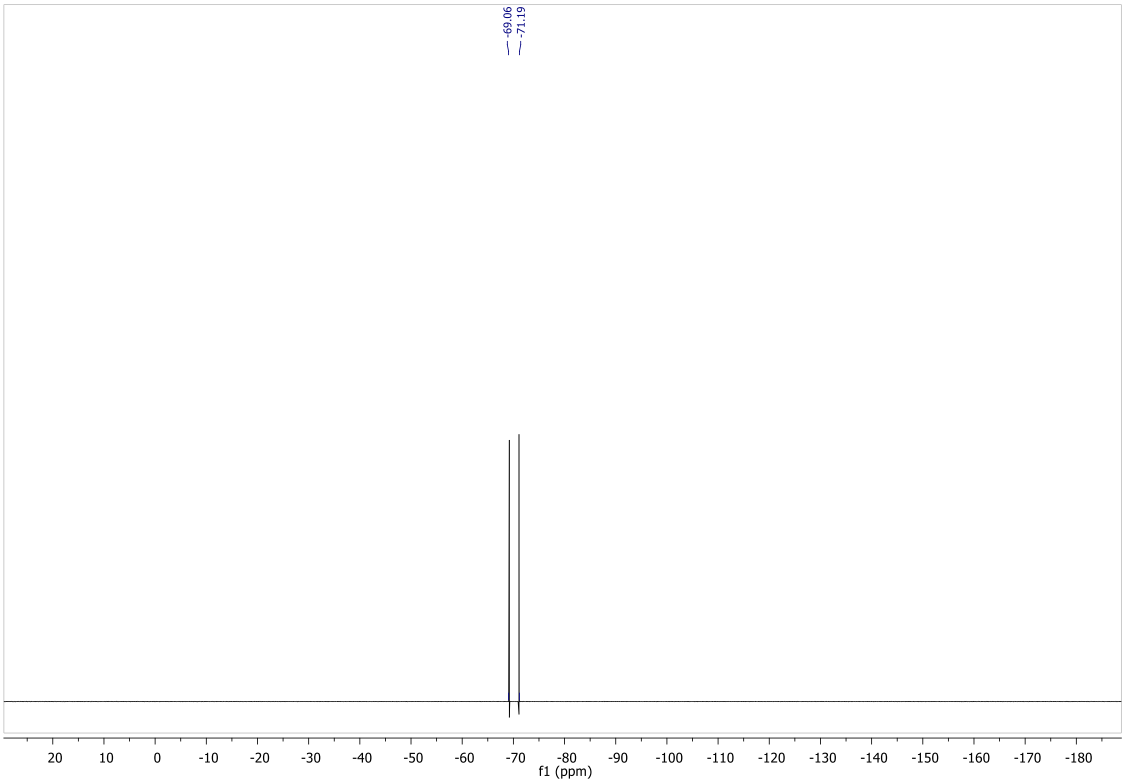

Figure S15. ^{19}F-NMR spectra of **4a** (DMSO-d_6).

^{31}P-NMR spectra of 4a

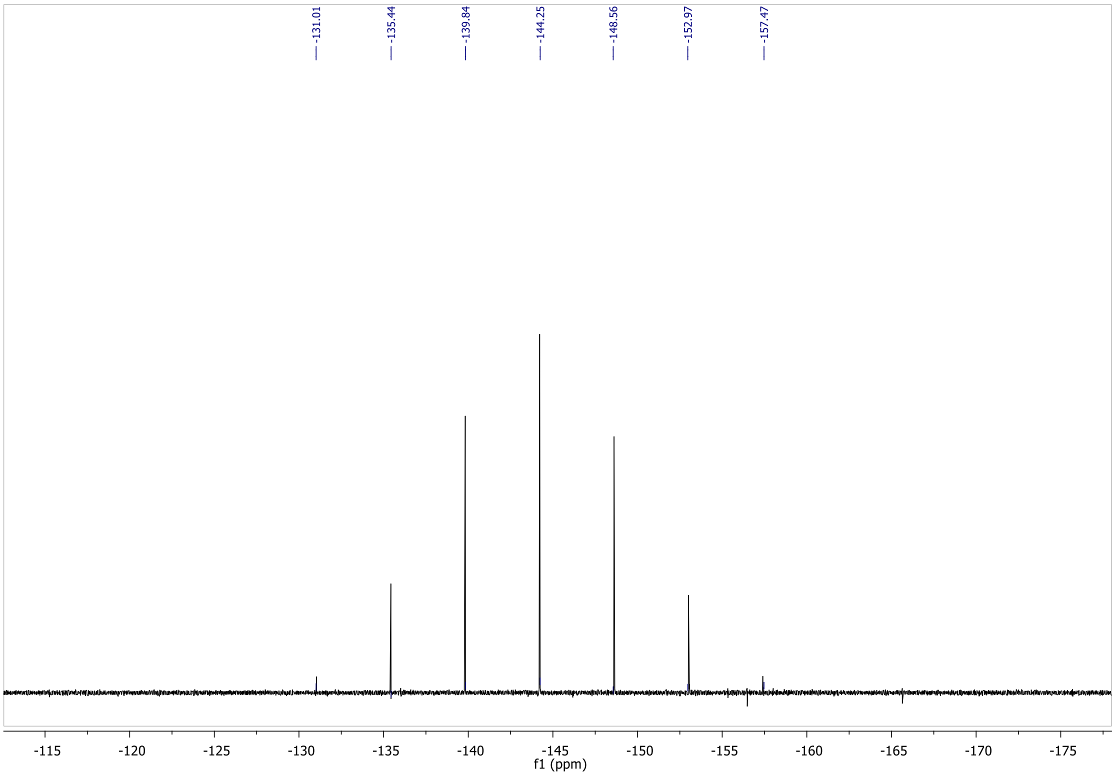

Figure S16. ^{31}P-NMR spectra of **4a** (DMSO-d_6).

^{1}H-NMR spectra of 4b

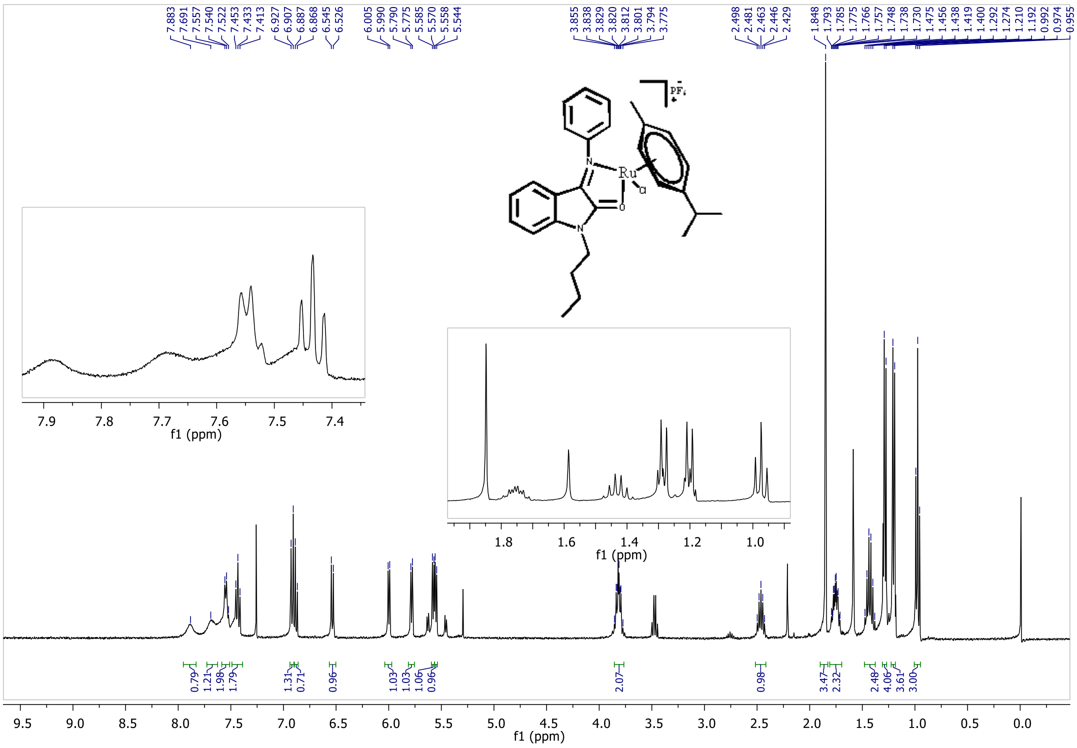

Figure S17. ^{1}H-NMR spectrum of **4b** ($CDCl_3$).

^{13}C-NMR spectra of 4b

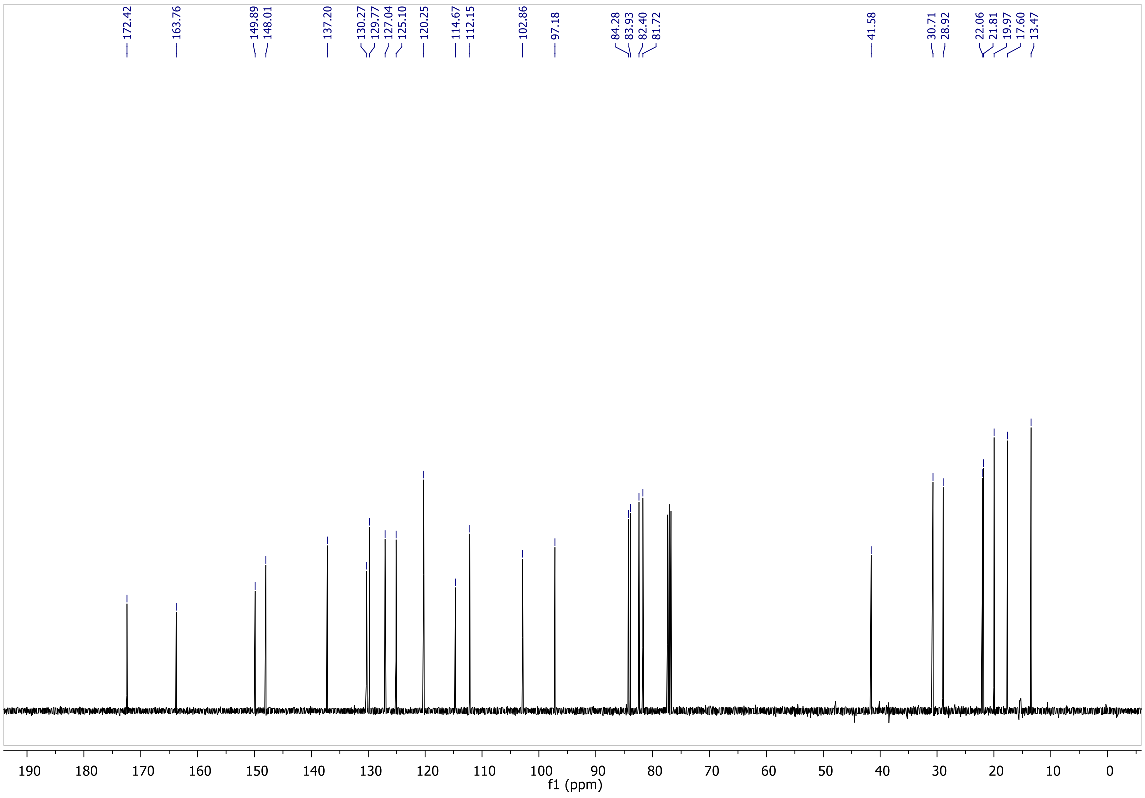

Figure S18. ^{13}C-NMR spectrum of **4b** ($CDCl_3$).

FTIR spectra of 4b

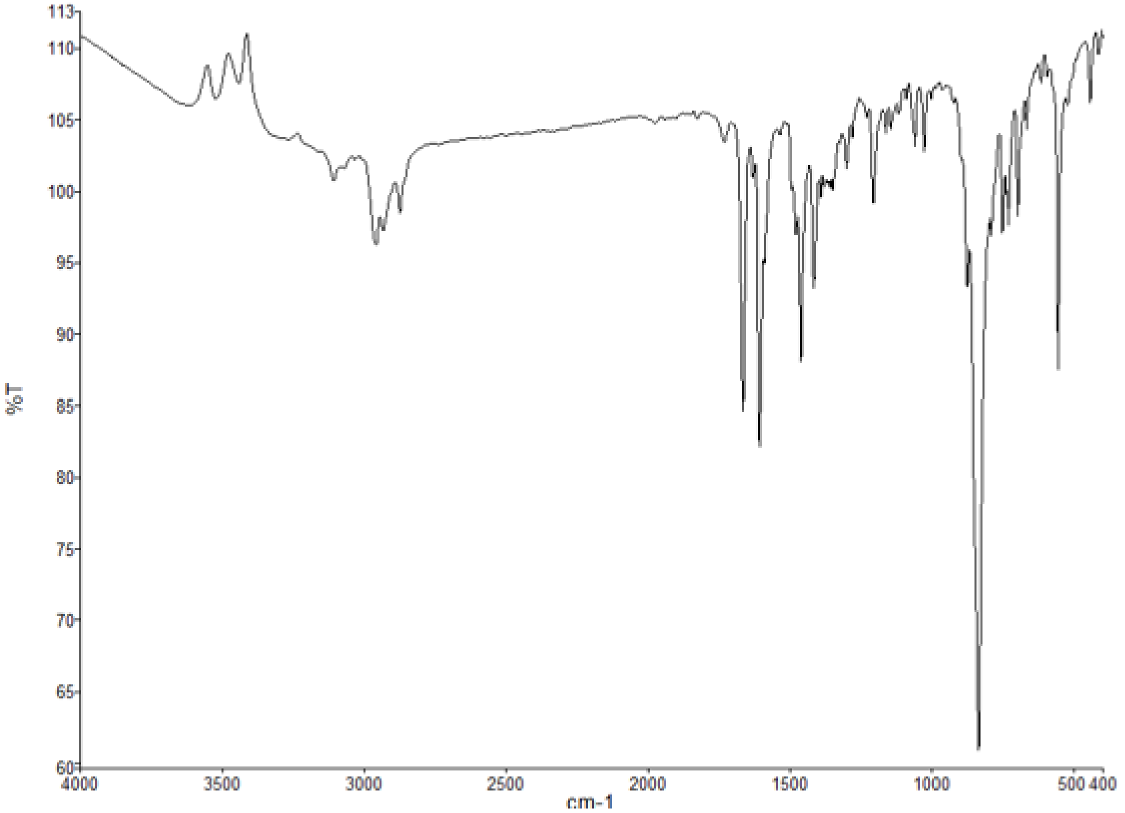

Figure S19. IR spectrum of **4b**.

^{19}F-NMR spectra of 4b

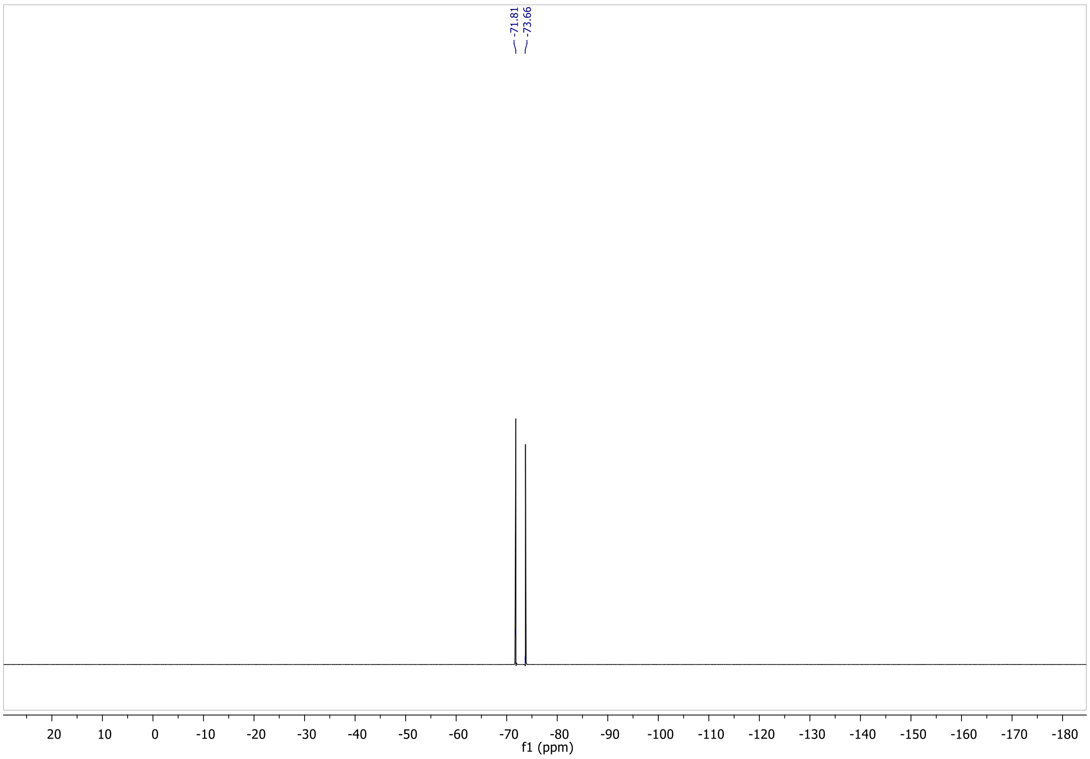

Figure S20. ^{19}F-NMR spectra of **4b** ($CDCl_3$).

^{31}P-NMR spectra of 4b

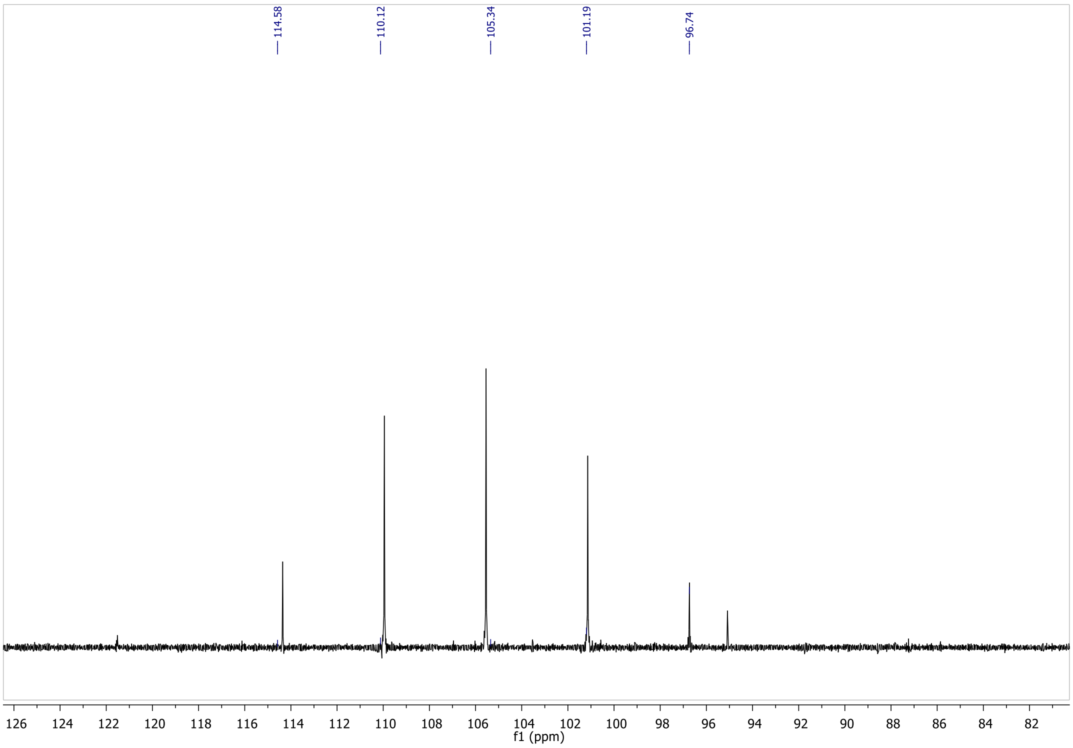

Figure S21. ^{31}P-NMR spectra of **4b** ($CDCl_3$).

^{1}H-NMR spectra of 4c

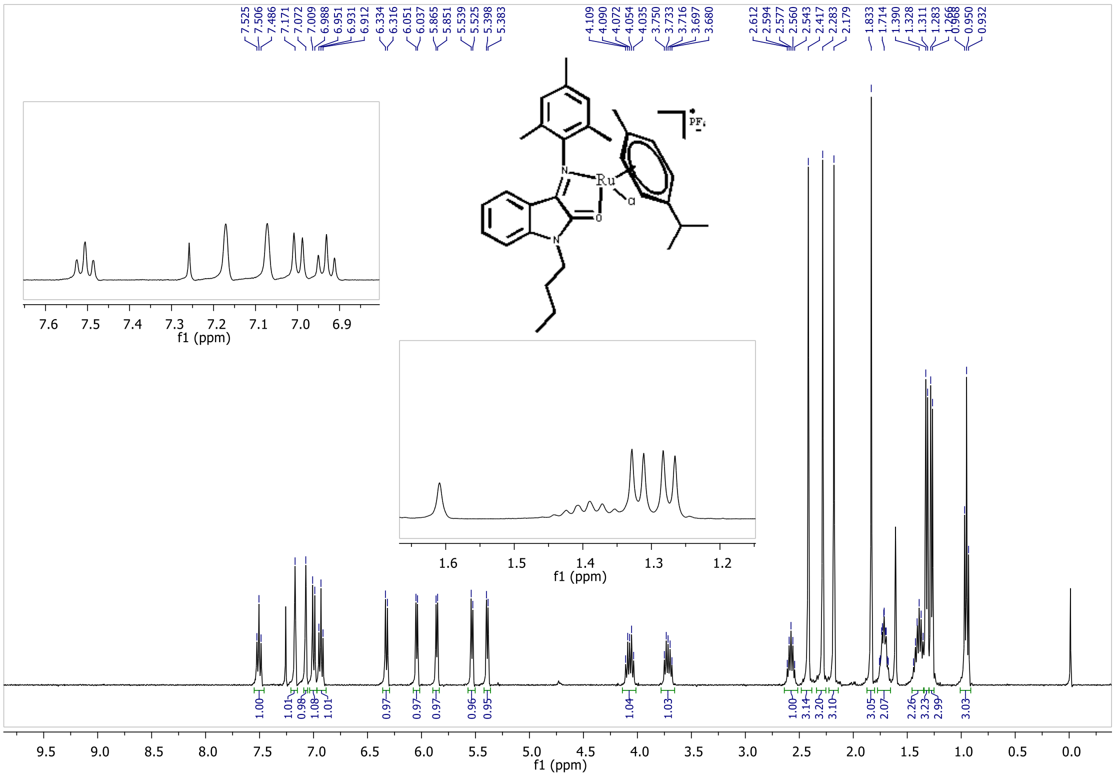

Figure S22. ^{1}H-NMR spectrum of **4c** ($CDCl_3$).

^{13}C-NMR spectra of 4c

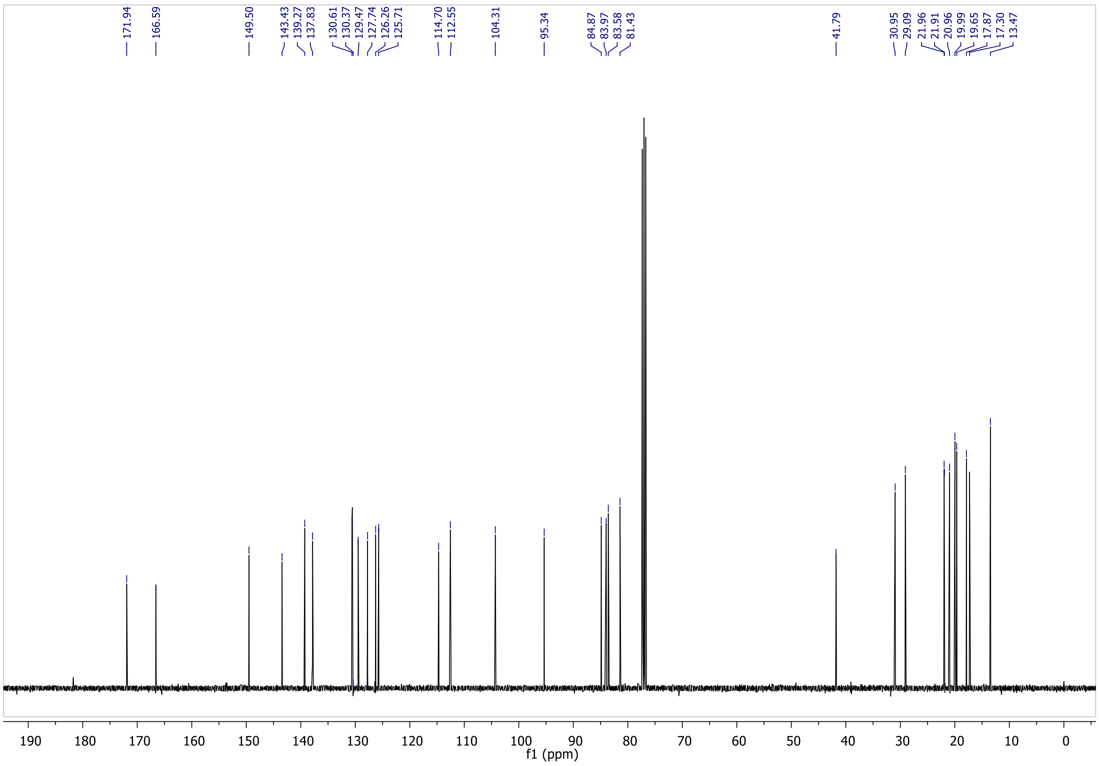

Figure S23. ^{13}C-NMR spectra of **4c** ($CDCl_3$).

FTIR spectra of 4c

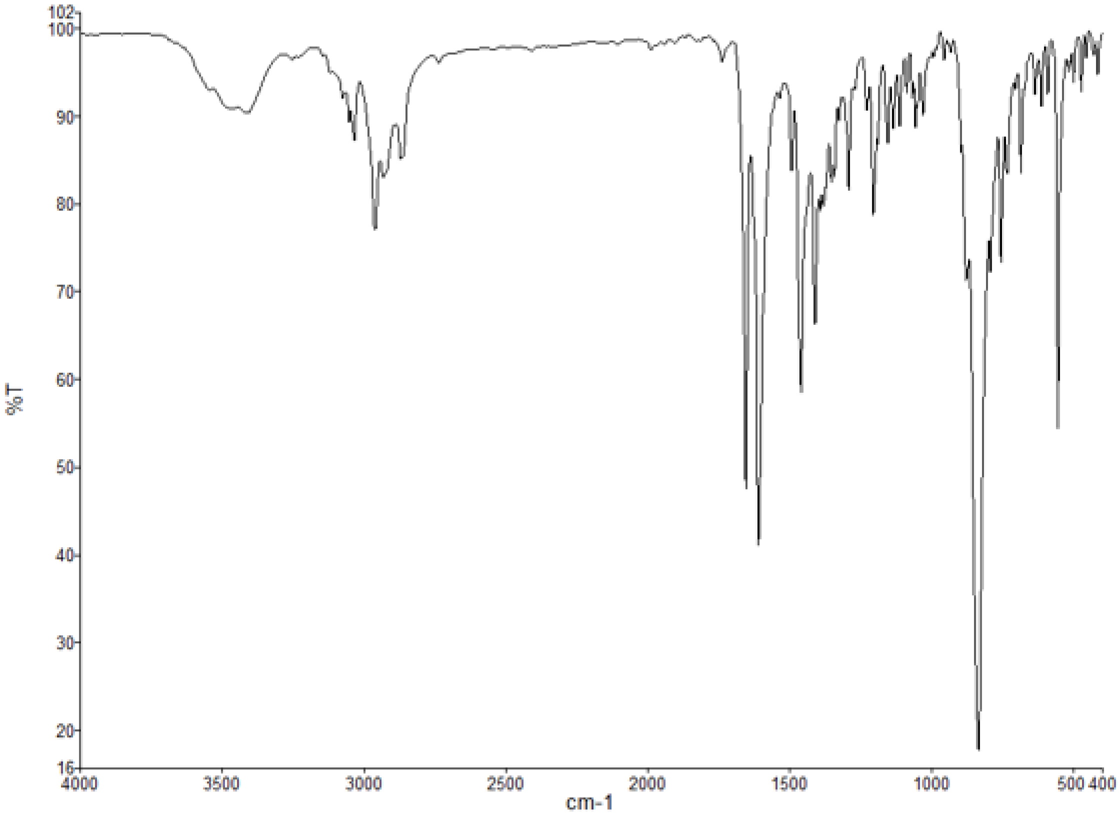

Figure S24. IR spectrum of **4c**.

^{19}F-NMR spectra of 4c

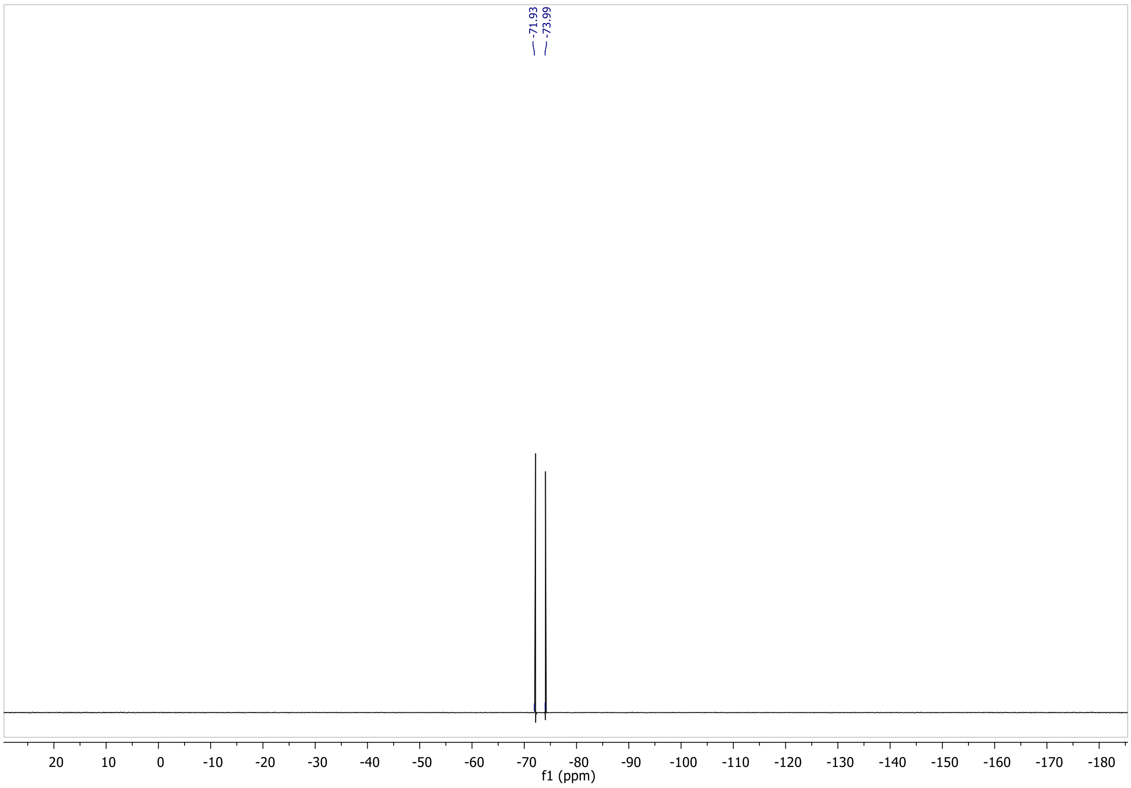

Figure S25. ^{19}F-NMR spectra of **4c** ($CDCl_3$).

^{31}P-NMR spectra of 4c

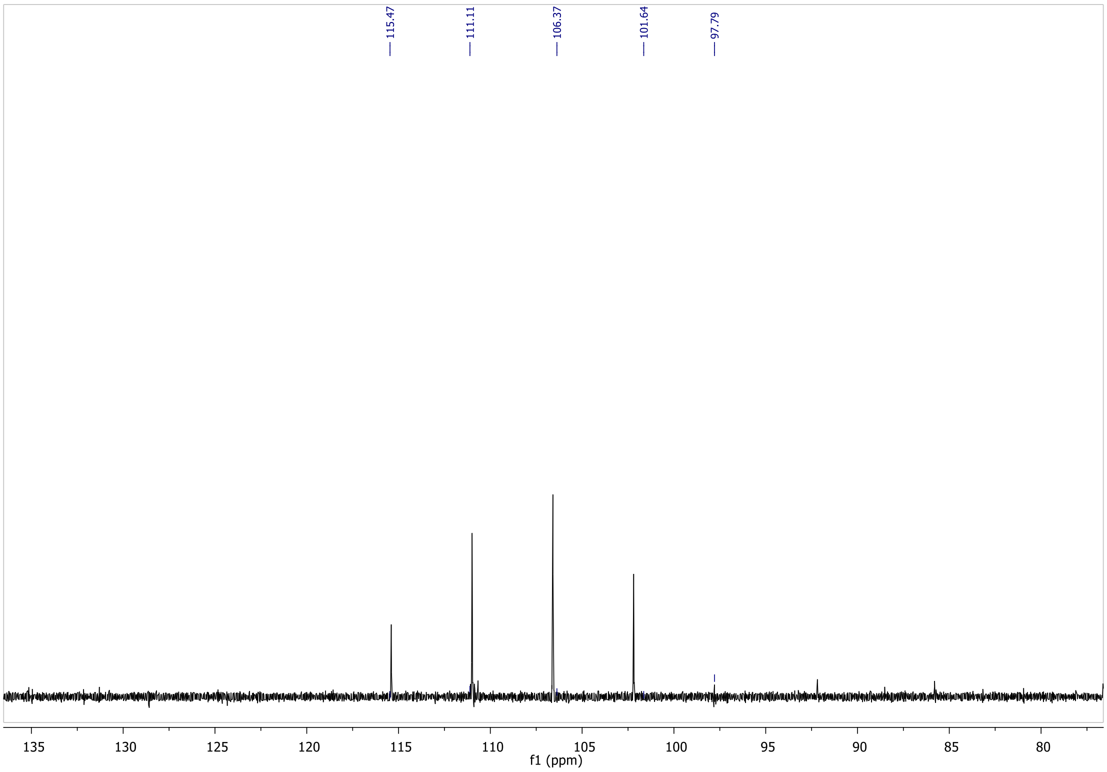

Figure S26. ^{31}P-NMR spectra of **4c** ($CDCl_3$).

^{1}H-NMR spectra of 4d

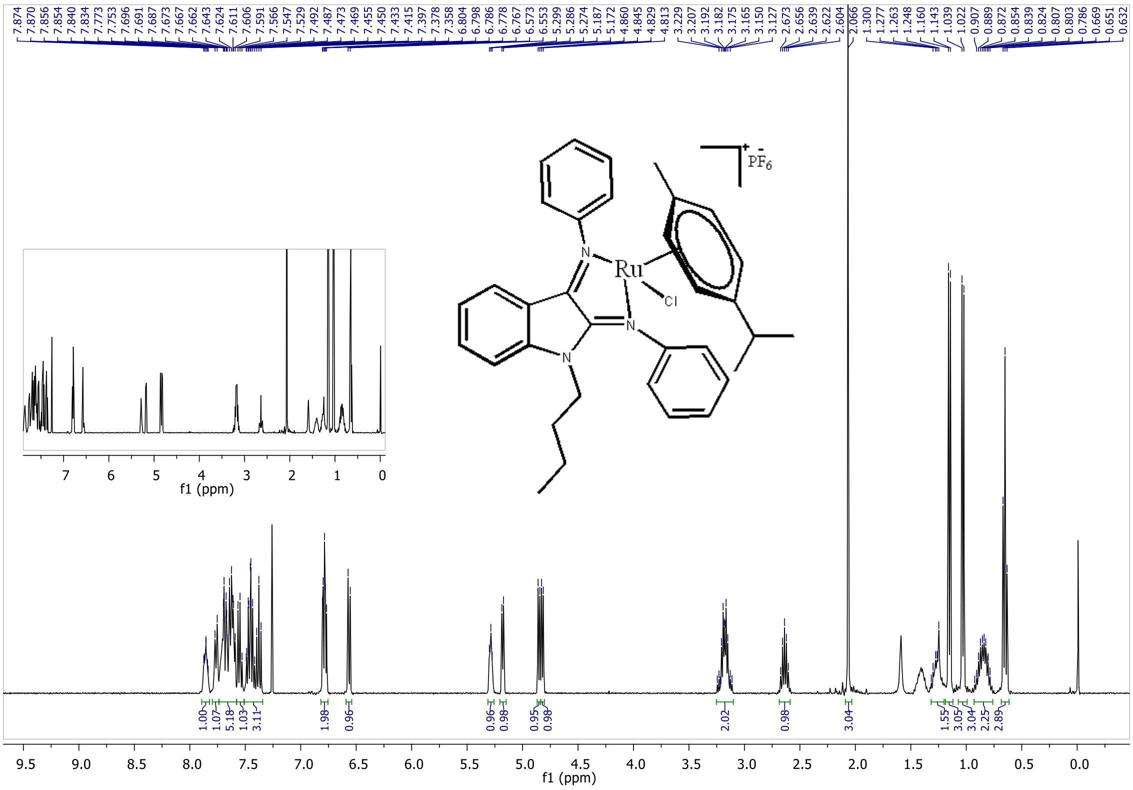

Figure S27. ^{1}H-NMR spectrum of **4d** ($CDCl_3$).

^{13}C-NMR spectra of 4d

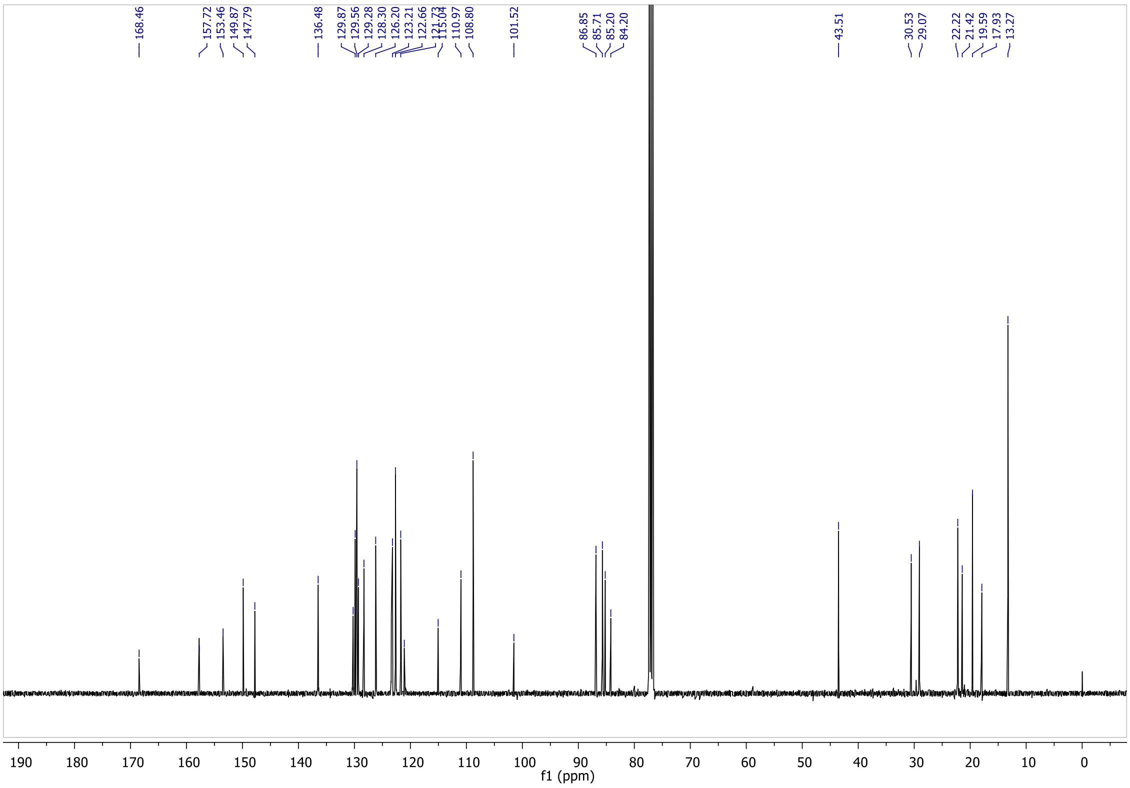

Figure S28. ^{13}C-NMR spectrum of **4d** ($CDCl_3$).

FTIR spectra of 4d

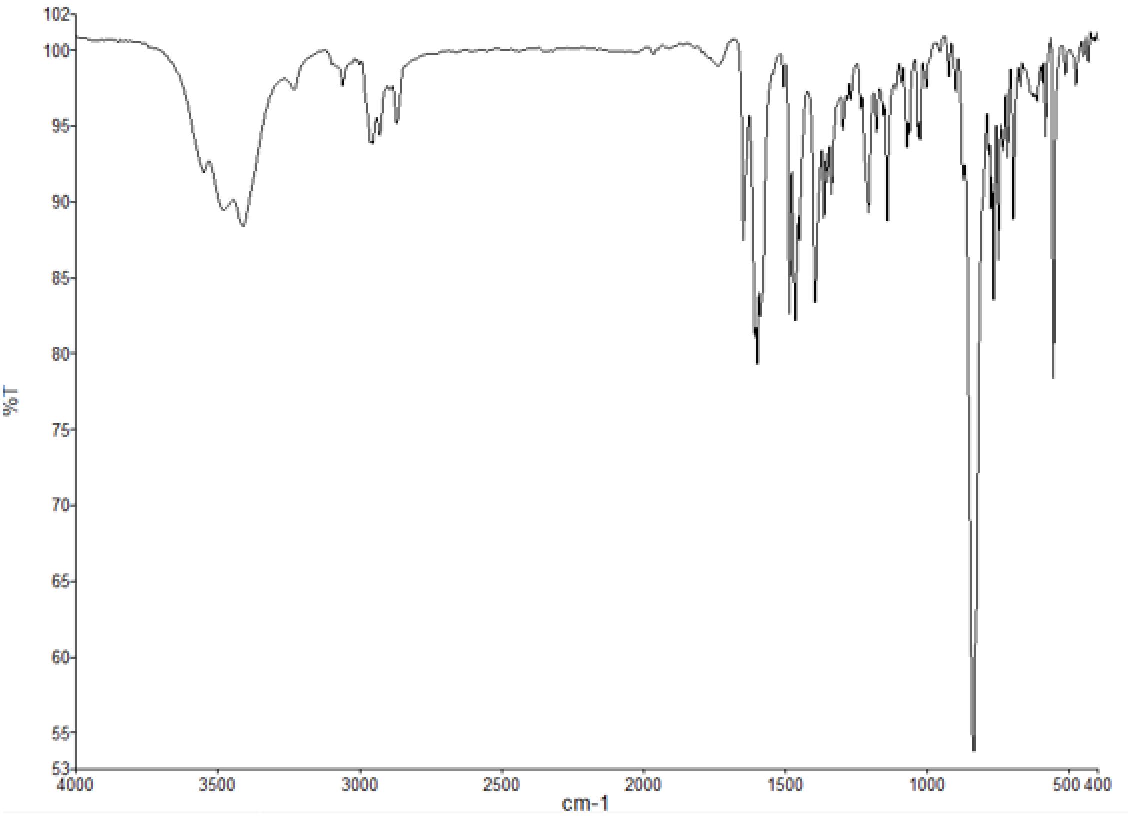

Figure S29. IR spectrum of **4d**.

^{19}F-NMR spectra of 4d

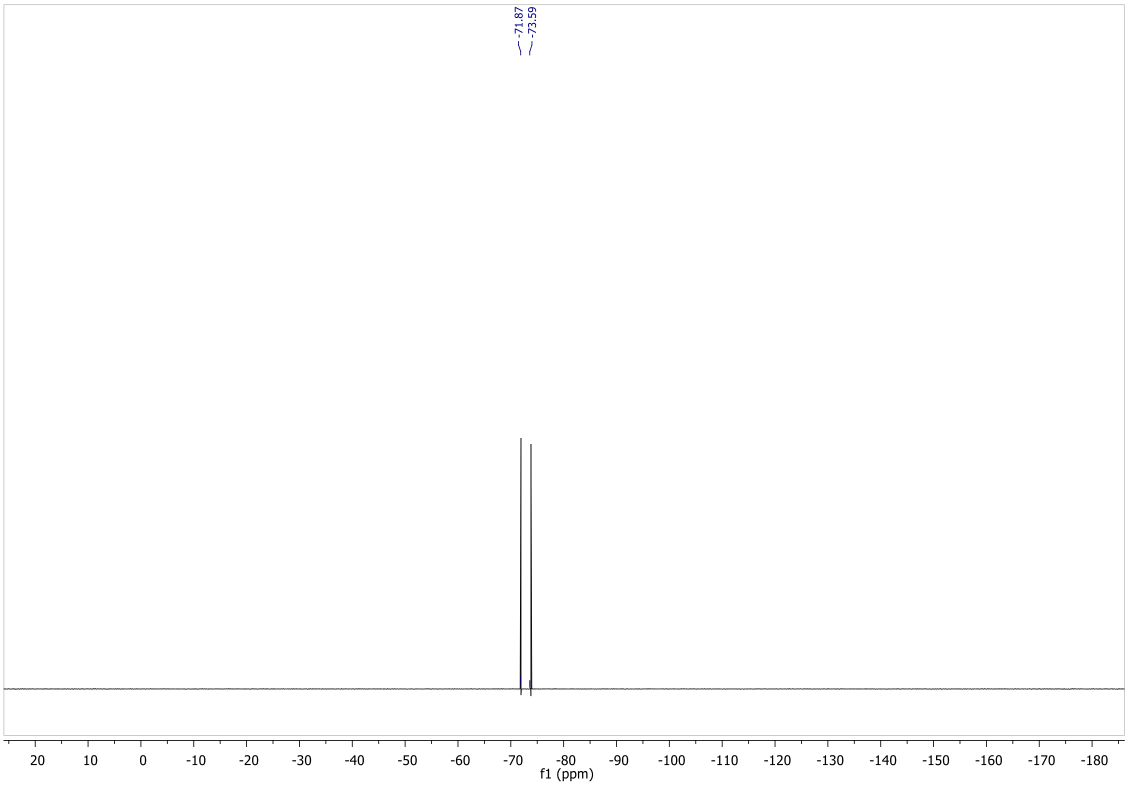

Figure S30. ^{19}F-NMR spectra of **4d** ($CDCl_3$).

^{31}P-NMR spectra of 4d

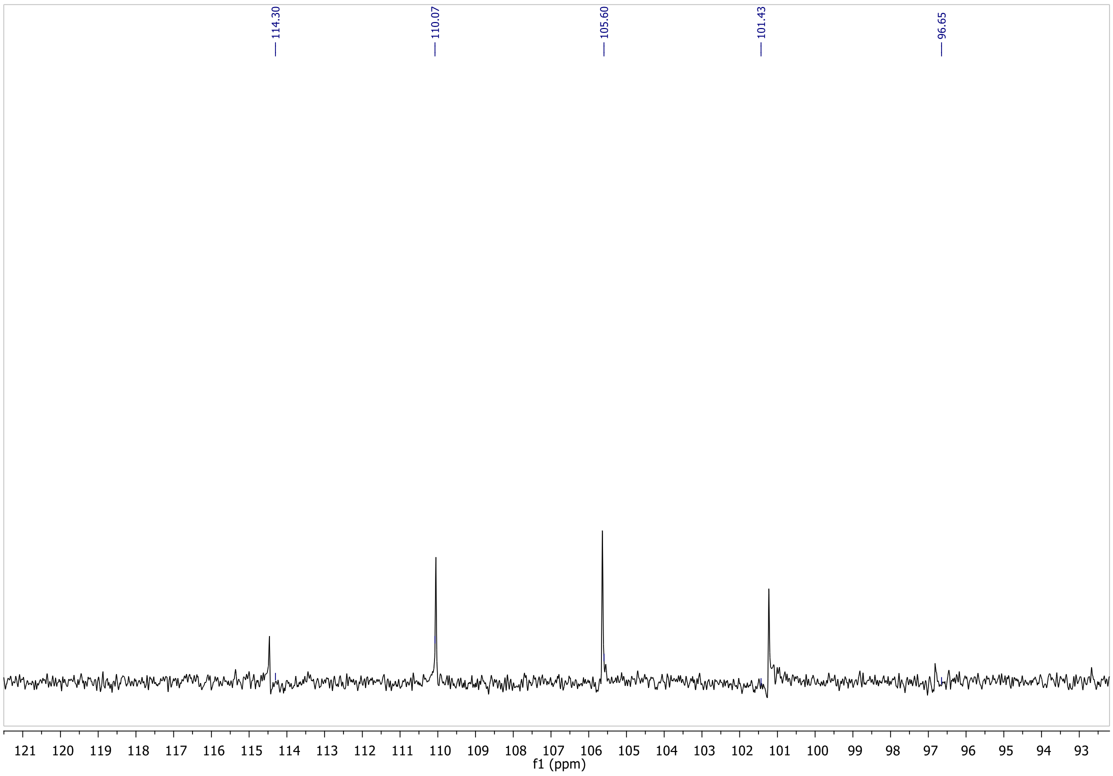

Figure S31. ^{31}P-NMR spectra of **4d** ($CDCl_3$).

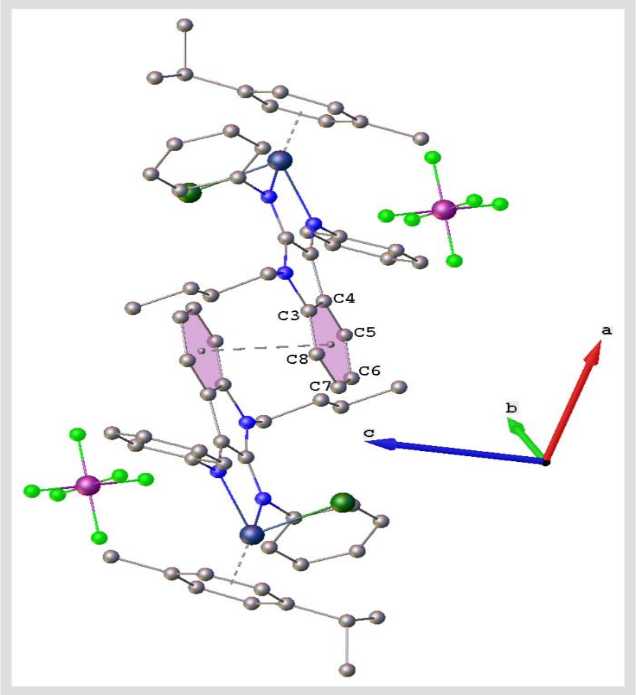

Figure S32. π···π stacking interaction in the crystal structure of complex **4d**. C3/C8 ring plane is shown with pink. Distance between the ring centroids is depicted with dashed lines. Hydrogen atoms are omitted for clarity.

Table S1. Crystal data and structure refinement parameters for complex **4d**.

Empirical formula	$C_{34}H_{37}ClN_3Ru, PF_6$
Formula weight	769.15
Crystal system	Monoclinic
Space group	$P\ 2_1/c$
a (Å)	16.8250(12)
b (Å)	10.135(5)
c (Å)	20.180(3)
β(°)	91.23(9)
V (Å^3)	3440.3(18)
Z	4
D_c (g cm^{-3})	1.485
θ range (º)	2.02-27.45
Data/restrain/parameter	7640/0/419
R_{int}	0.251
Goodness of fit (F^2)	0.916
R_1/wR_2 [$I > 2\sigma(I)$]	0.086/ 0.1196
R_1/wR_2 (all data)	0.232/ 0.162
$\Delta\rho_{max}/\Delta\rho_{min}$ (eÅ^{-3})	-0.715/ 0.783
CCDC number	2288204

Figure S33. Cell viability results of the ligands **2, 3a-c** in HepG2, MCF7, PC-3 and HEK-293 cell lines after 24, 48 and 72 h of exposure.

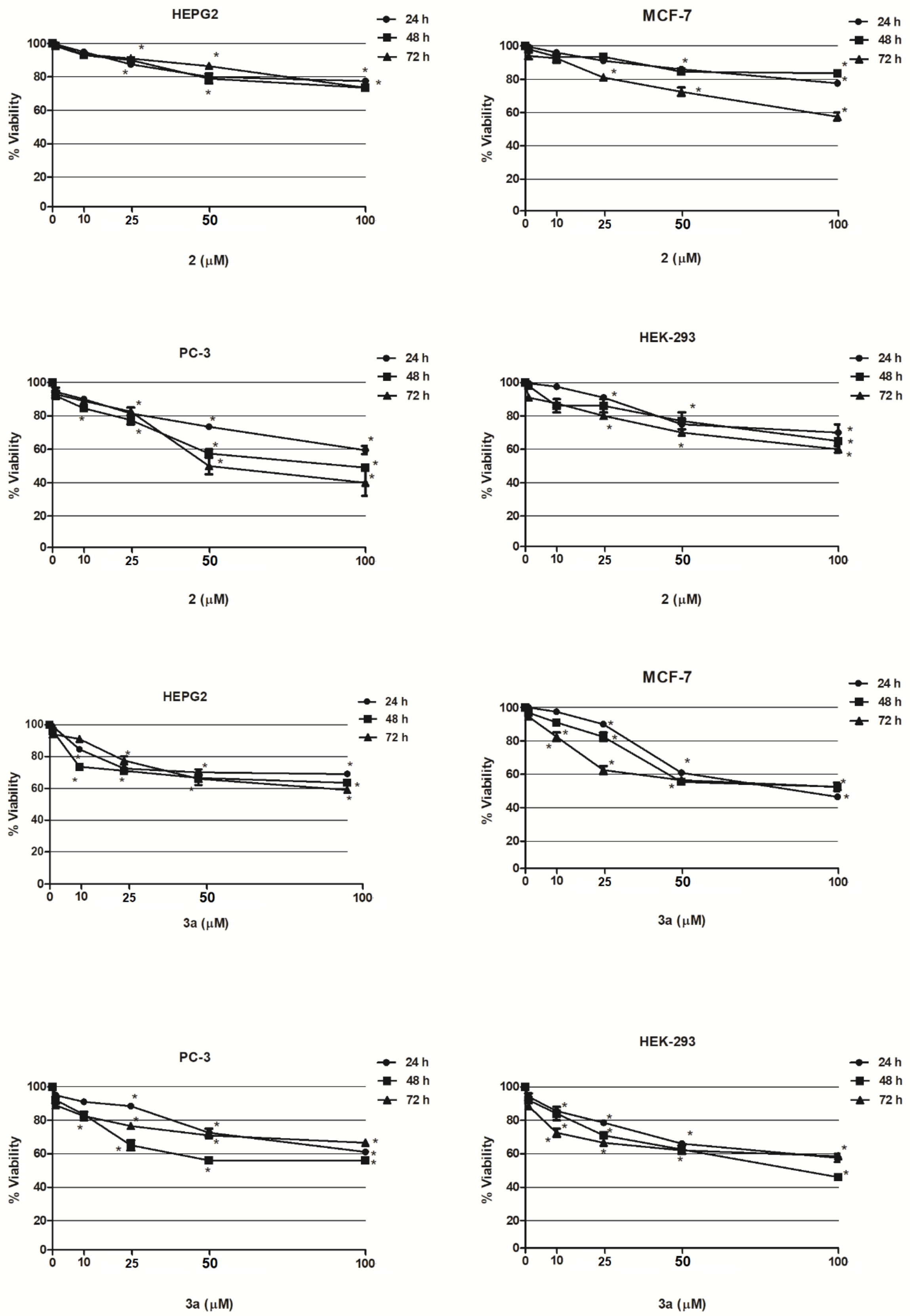

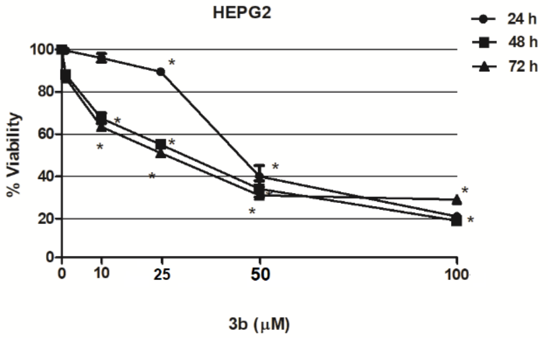
HEPG2
24 h
48 h
72 h
% Viability
3b (μM)

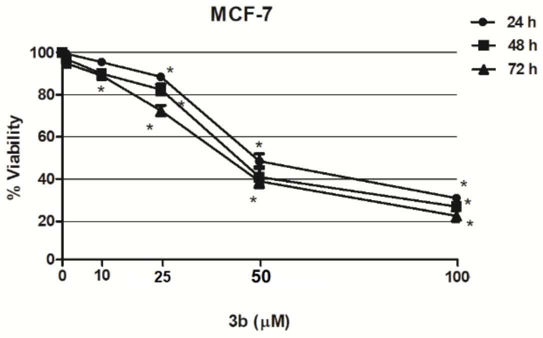
MCF-7
24 h
48 h
72 h
% Viability
3b (μM)

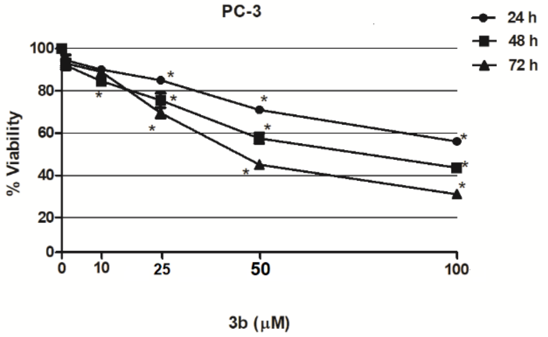
PC-3
24 h
48 h
72 h
% Viability
3b (μM)

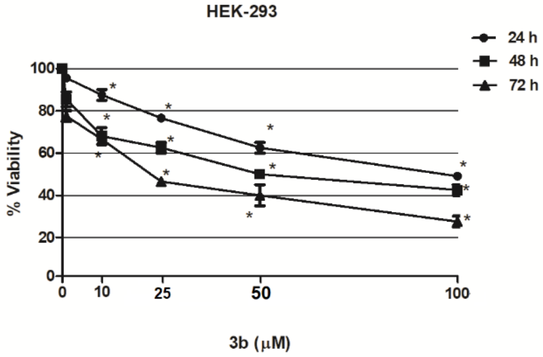
HEK-293
24 h
48 h
72 h
% Viability
3b (μM)

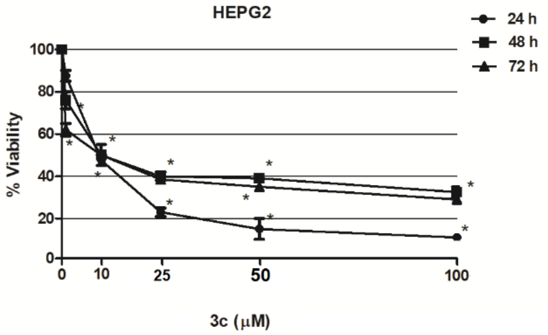
HEPG2
24 h
48 h
72 h
% Viability
3c (μM)

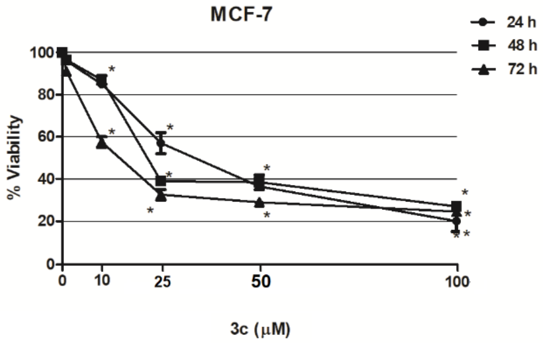
MCF-7
24 h
48 h
72 h
% Viability
3c (μM)

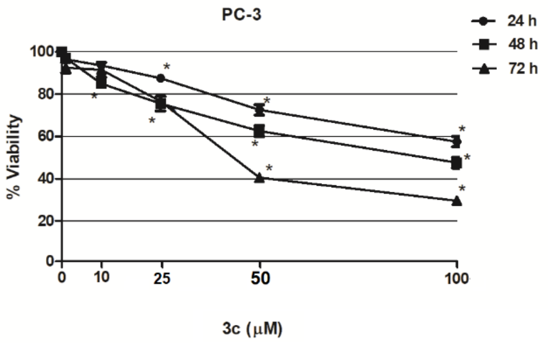
PC-3
24 h
48 h
72 h
% Viability
3c (μM)

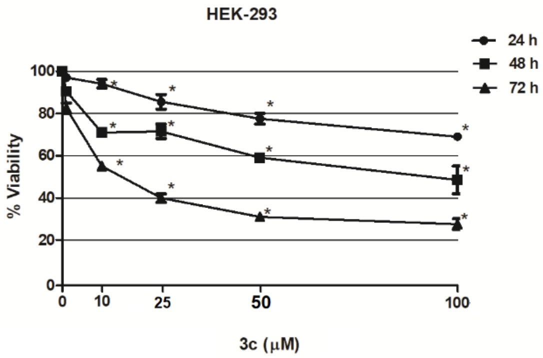
HEK-293
24 h
48 h
72 h
% Viability
3c (μM)

Figure S34. Cell viability results of the **4a-c** complexes in HepG2, MCF7, PC-3 and HEK-293 cell lines after 24, 48 and 72 h of exposure.

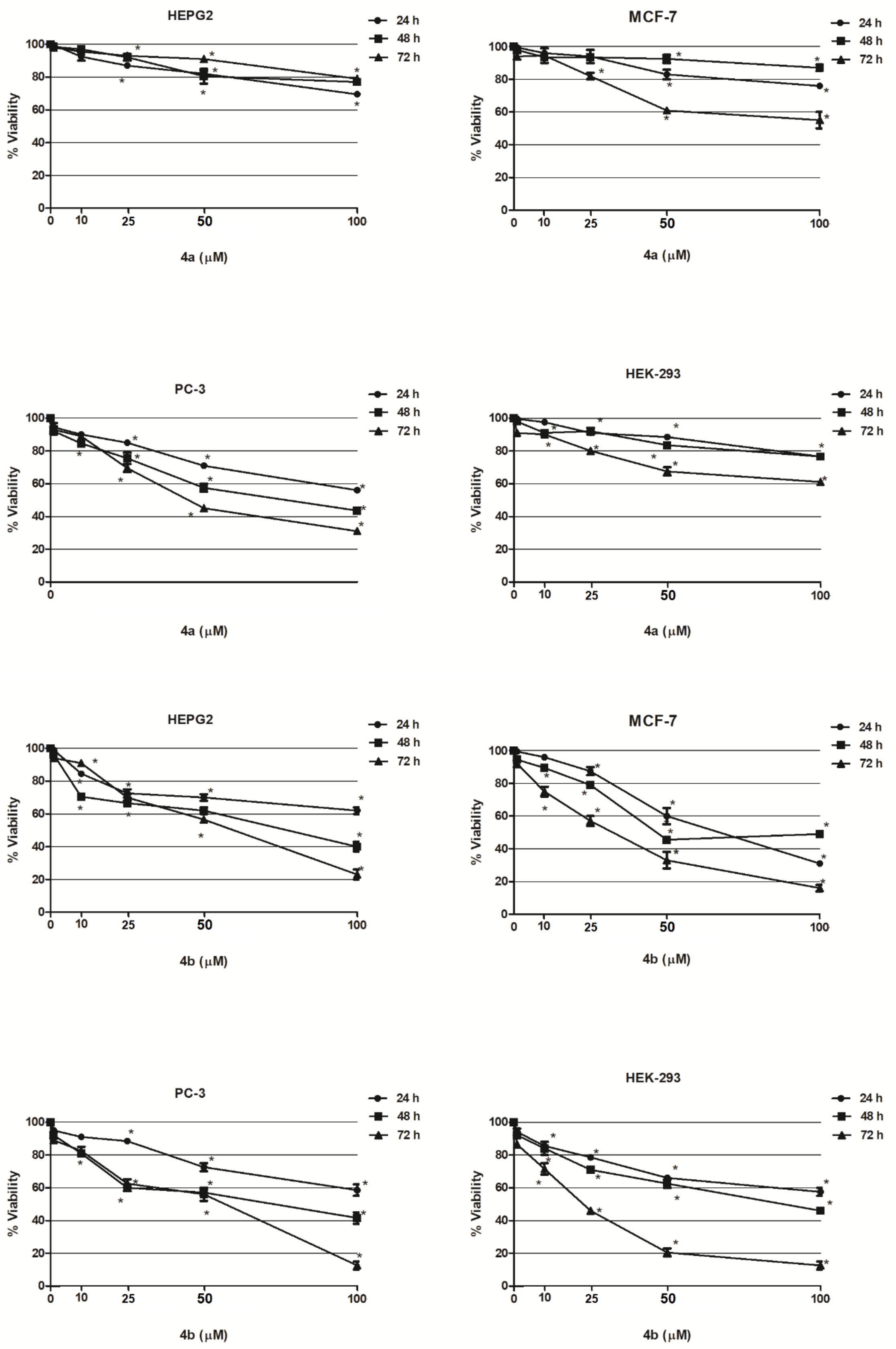

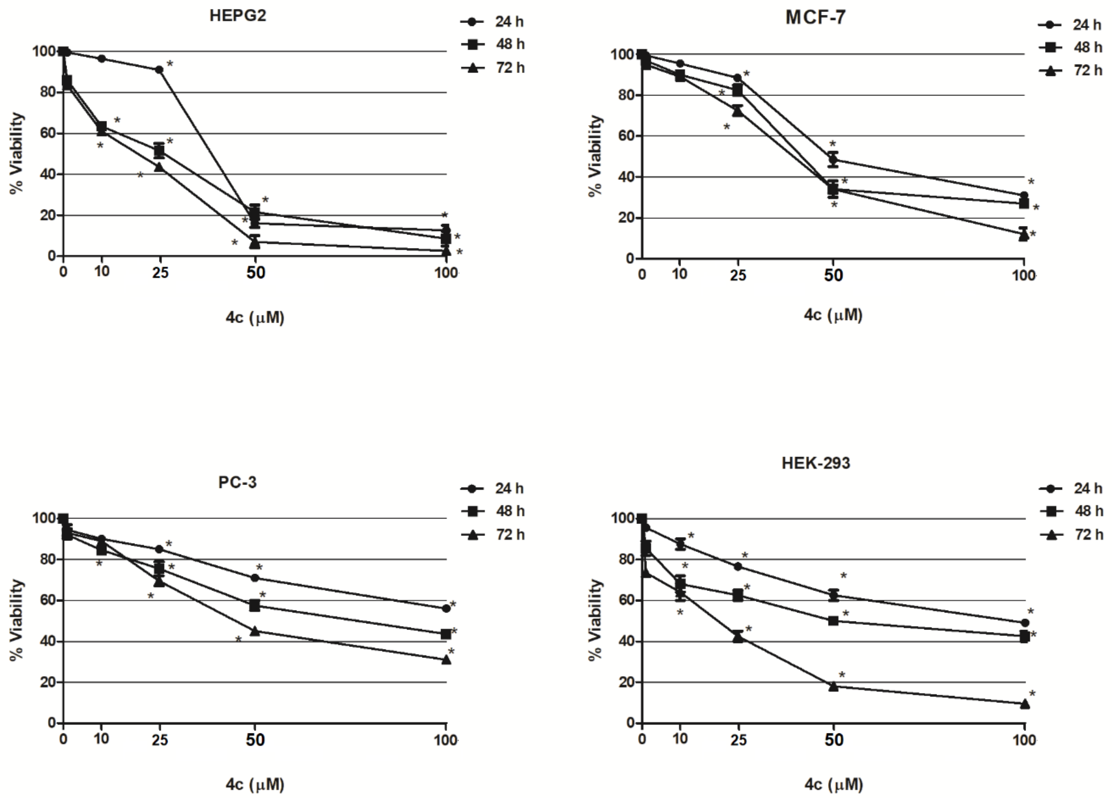
HEPG2
MCF-7
PC-3
HEK-293
24 h
48 h
72 h
% Viability
4c (μM)
0
10
25
50
100
20
40
60
80

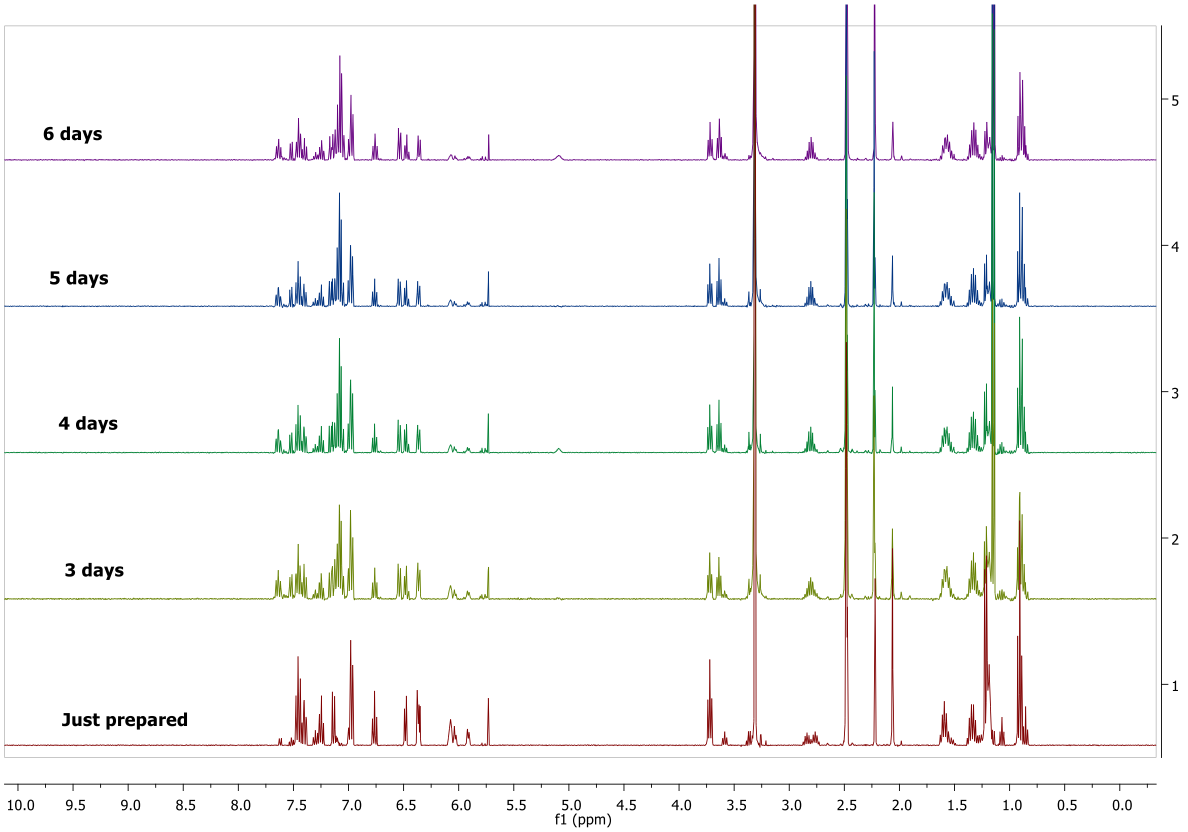

Figure S35. The stability of complex **4b** was tracked via ^{1}H NMR spectroscopy in DMSO-d_6 over 6 days.

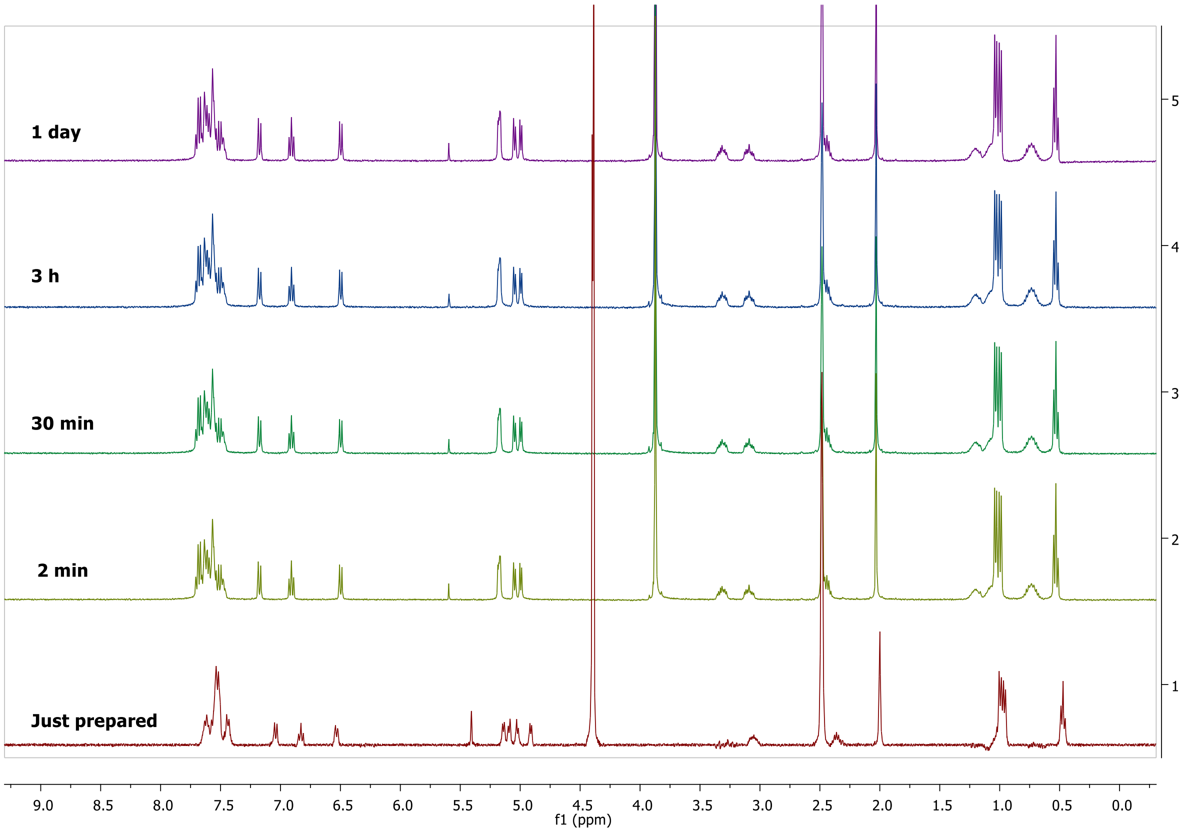

Figure S36. The stability of complex **4d** was tracked via ^{1}H NMR spectroscopy in D_2O/DMSO-d_6 (20:80) over 1 day.

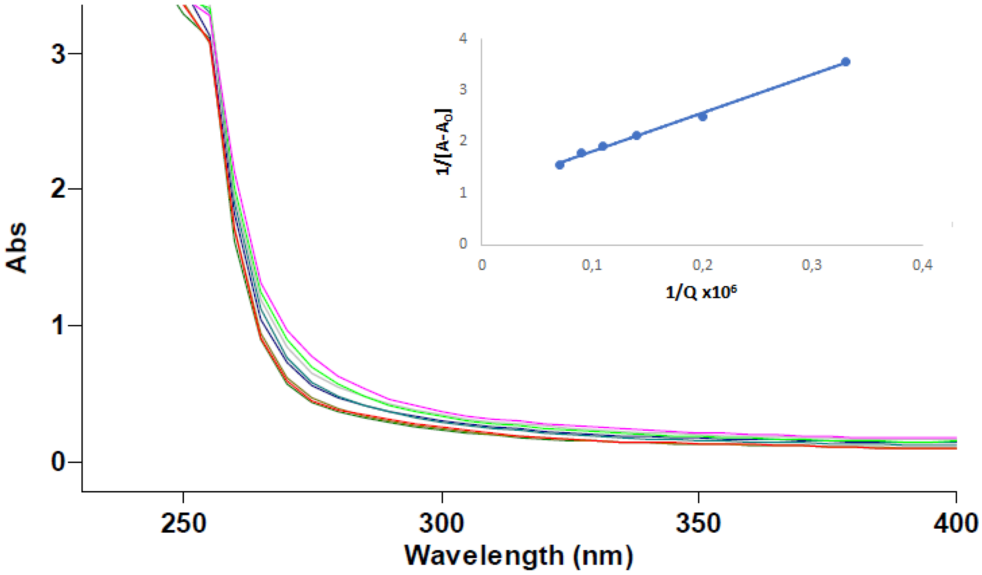

Figure S37. UV-Vis spectra of FS-DNA with different concentrations of **4d** K_b was calculated by the ratio of intercept and slope of plot between 1/(A_0-A) and 1/[Q].
